# The genome of *Prasinoderma coloniale* unveils the existence of a third phylum within green plants

**DOI:** 10.1038/s41559-020-1221-7

**Published:** 2020-06-22

**Authors:** Linzhou Li, Sibo Wang, Hongli Wang, Sunil Kumar Sahu, Birger Marin, Haoyuan Li, Yan Xu, Hongping Liang, Zhen Li, Shifeng Cheng, Tanja Reder, Zehra Çebi, Sebastian Wittek, Morten Petersen, Barbara Melkonian, Hongli Du, Huanming Yang, Jian Wang, Gane Ka-Shu Wong, Xun Xu, Xin Liu, Yves Van de Peer, Michael Melkonian, Huan Liu

**Affiliations:** 1 State Key Laboratory of Agricultural Genomics, BGI-Shenzhen, Shenzhen, China; 2 Department of Biotechnology and Biomedicine, Technical University of Denmark, Lyngby, Denmark; 3 Department of Biology, University of Copenhagen, Copenhagen, Denmark; 4 BGI Education Center, University of Chinese Academy of Sciences, Shenzhen, China; 5 Institute for Plant Sciences, Department of Biological Sciences, University of Cologne, Cologne, Germany; 6 Department of Plant Biotechnology and Bioinformatics (Ghent University) and Center for Plant Systems Biology, Ghent, Belgium; 7 Central Collection of Algal Cultures, Faculty of Biology, University of Duisburg-Essen, Essen, Germany; 8 School of Biology and Biological Engineering, South China University of Technology, Guangzhou, China; 9 Department of Biological Sciences and Department of Medicine, University of Alberta, Edmonton, Alberta Canada; 10 Guangdong Provincial Key Laboratory of Genome Read and Write, BGI-Shenzhen, Shenzhen, China; 11 College of Horticulture, Nanjing Agricultural University, Nanjing, China; 12 Centre for Microbial Ecology and Genomics, Department of Biochemistry, Genetics and Microbiology, University of Pretoria, Pretoria, South Africa

**Keywords:** Evolutionary genetics, Molecular evolution, Phylogenetics, Taxonomy, Evolutionary biology

## Abstract

Genome analysis of the pico-eukaryotic marine green alga *Prasinoderma coloniale* CCMP 1413 unveils the existence of a novel phylum within green plants (Viridiplantae), the Prasinodermophyta, which diverged before the split of Chlorophyta and Streptophyta. Structural features of the genome and gene family comparisons revealed an intermediate position of the *P. coloniale* genome (25.3 Mb) between the extremely compact, small genomes of picoplanktonic Mamiellophyceae (Chlorophyta) and the larger, more complex genomes of early-diverging streptophyte algae. Reconstruction of the minimal core genome of Viridiplantae allowed identification of an ancestral toolkit of transcription factors and flagellar proteins. Adaptations of *P. coloniale* to its deep-water, oligotrophic environment involved expansion of light-harvesting proteins, reduction of early light-induced proteins, evolution of a distinct type of C_4_ photosynthesis and carbon-concentrating mechanism, synthesis of the metal-complexing metabolite picolinic acid, and vitamin B_1_, B_7_ and B_12_ auxotrophy. The *P. coloniale* genome provides first insights into the dawn of green plant evolution.

## Abstract (web summary)

Genome analysis of the pico-eukaryotic marine green alga *Prasinoderma coloniale* CCMP 1413 unveils the existence of a novel phylum within green plants (Viridiplantae), the Prasinodermophyta, which diverged before the split of Chlorophyta and Streptophyta.

## Main

One of the most important biological events in the history of life was the successful colonization of the terrestrial landscape by green plants (Viridiplantae) that paved the way for terrestrial animal evolution, altering geomorphology and changes in the Earth’s climate^1–3^. The Viridiplantae comprise perhaps 500,000 species, ranging from the smallest to the largest eukaryotes^4,5^. Divergence time estimates from molecular data suggest that Viridiplantae may be close to 1 billion years old^6,7^. All extant green plants are classified in either of two divisions/phyla, Chlorophyta and Streptophyta, which differ structurally, biochemically and molecularly^8–12^. The Streptophyta contain the land plants (embryophytes) and a paraphyletic assemblage of algae known as the streptophyte algae, whereas all other green algae comprise the Chlorophyta. The reconstruction of phylogenetic relationships across green plants using transcriptomic or genomic data provided evidence that unicellular, often scaly, flagellate organisms were positioned near the base of the radiation in both phyla^13–16^, corroborating earlier proposals based on ultrastructural analyses that the common ancestor of all green plants may have been a scaly flagellate^17,18^. The search for an extant relative of such a flagellate, however, has been in vain, although an initial report suggested that *Mesostigma viride* diverged before the split of Chlorophyta and Streptophyta^19^, a result not corroborated by later studies^20^. *M. viride* is now recognized as an early-diverging member of the Streptophyta^21,22^. While the majority of the early-diverging lineages in the Chlorophyta consisted of (mostly marine) scaly flagellates, some lineages were represented by very small, non-flagellate unicells often surrounded by cell walls^23,24^. One of these lineages, provisionally termed ‘Prasinococcales’^23^ (clade VI), could not be reliably positioned in phylogenetic trees^24,25^. A major step forward was made when it was discovered that an enigmatic, non-cultured group of deep-water, oceanic macroscopic algae of palmelloid organization comprising the genera *Verdigellas* and *Palmophyllum* formed a deeply diverging lineage of Viridiplantae that included the Prasinococcales^26^. Later, the class Palmophyllophyceae was established for these organisms as the first divergence in Chlorophyta, that is sister to all other Chlorophyta^27^. Phylogenies based on nuclear-encoded ribosomal RNA genes (4,579 positions), however, placed Palmophyllophyceae as the earliest divergence in Viridiplantae, but monophyly of Chlorophyta + Streptophyta to the exclusion of Palmophyllophyceae, received no support in these analyses^27^.

**Fig. 1:**
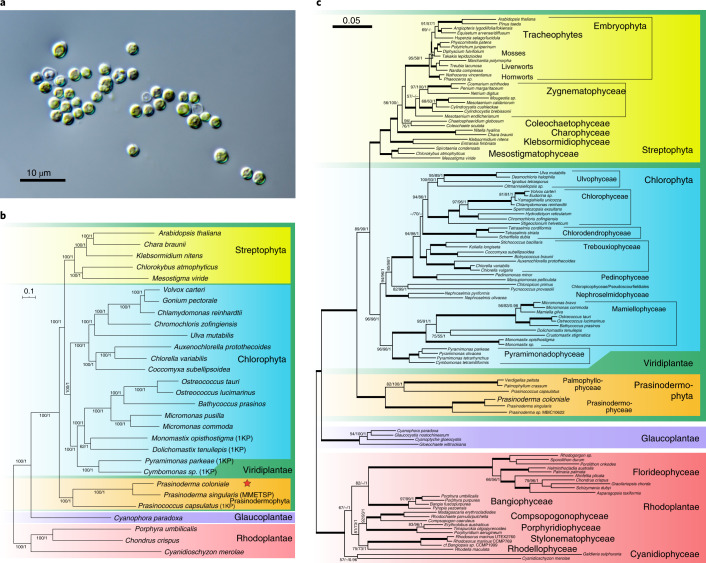
Phylogenetic analysis of *P. coloniale*. **a**, Light micrograph of *P. coloniale*. **b**, The phylogenetic tree was constructed using the maximum-likelihood method in RAxML and MrBayes based on a concatenated sequence alignment of 256 single-copy genes (500 bootstraps). **c**, The basal divergence of the new phylum Prasinodermophyta, as revealed by analyses of complete nuclear- and plastid-encoded rRNA operons from 109 Archaeplastida. The rRNA dataset comprised 8,818 aligned positions and contained representatives of all major lineages of Rhodoplantae (seven classes), Glaucoplantae (four genera) and Viridiplantae (three divisions with several classes) including embryophytes. Shown is the RAxML phylogeny (GTRGAMMA model); the three support values at branches are RAxML/IQ-TREE bootstrap percentages/Bayesian posterior probabilities. Bold branches received maximal support (100/100/1).

To date, genomic resources for the Palmophyllophyceae have been limited to organelle genomes. Here we present the first nuclear genome sequence of a unicellular member of this lineage, *Prasinoderma coloniale* (Fig. 1a). Based on phylogenomic analyses, we establish a new phylum for this group, the Prasinodermophyta, with two classes, as the earliest divergence of the Viridiplantae. The genome of *P. coloniale* provided new insights into pico-eukaryotic biology near the dawn of green plant evolution.

## Results and discussion

### Genome sequencing and characteristics

**Extended Data Fig. 1:**
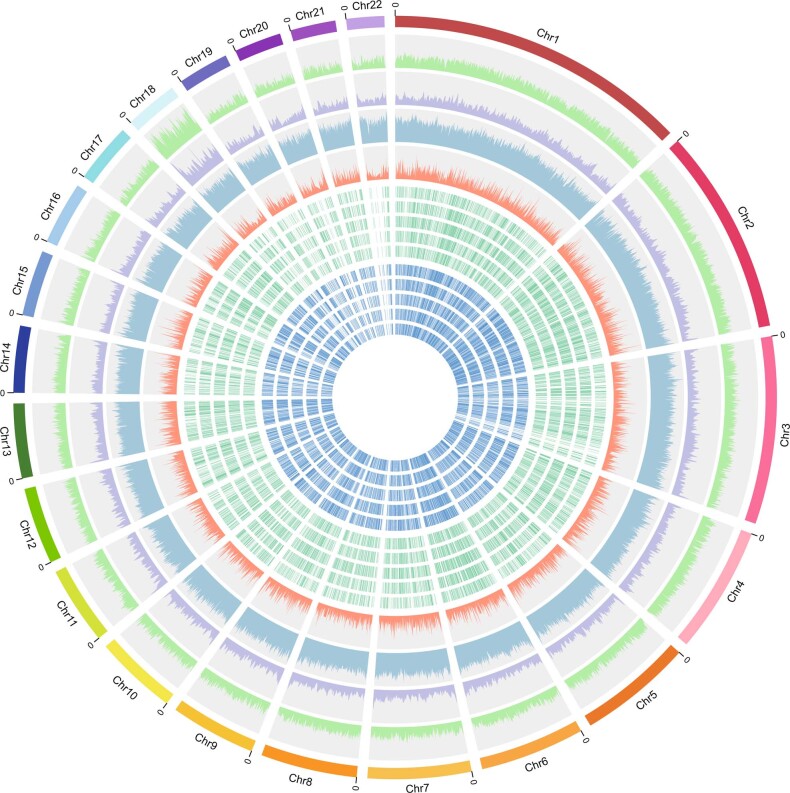
A physical map of the *P. coloniale* genome. Outer ring: The 22 chromosomes were labeled from Chr1 to Chr22. Inner rings 2–5 (from outside to inside): Illumina sequencing depth colored in light green (y-axis min-max: 0–592). PacBio sequencing depth colored in light purple (y-axis min-max: 0–67). GC content of *P. coloniale* chromosomes in light blue (y-axis min-max: 0–80.0). The gene number distribution of *P. coloniale* colored in red (y-axis min-max: 0–38). The slide window of inner rings 2–5 is 5,000 bp. Inner rings 6–15: Genes shared between *P. coloniale* and other early-diverging viridiplant genomes, from outside to inside. *M. viride, C. atmophyticus, K. nitens, C. braunii* and *M. endlicherianum* (green), *M. commoda, M. pusilla, B. prasinos, O. lucimarinus* and *O. tauri* (blue).

The genome size of *P. coloniale* was estimated to be about 26.04 Mb. After reads filtering (7.4 Gb PacBio data) and self-correction, a 25.3 Mb genome was de novo assembled consisting of 22 chromosomes, including the complete chloroplast and mitochondrial genomes (Supplementary Figs. 1 and 2). The sizes of the individual chromosomes varied from 0.45 to 3.60 Mb. BUSCO analysis showed a high degree of completeness of the genome, with 282 out of 303 (93.1%) complete eukaryotic universal genes (Supplementary Table 1). Additionally, 99.38% (Supplementary Table 2) of the transcriptome could be mapped to the assembled genome. *P. coloniale* has a GC content of 69.8%, while 6.51% of the genome consists of repeats (Supplementary Fig. 3, Supplementary Table 3 and Extended Data Fig. 1). A total of 7,139 protein-coding genes were annotated, of which 6,996 were supported by the transcriptome. Additionally, 6,759 (94.7%) genes were annotated from known protein databases (Supplementary Table 4).

### Phylogenetic analyses and Prasinodermophyta div. nov

**Extended Data Fig. 2:**
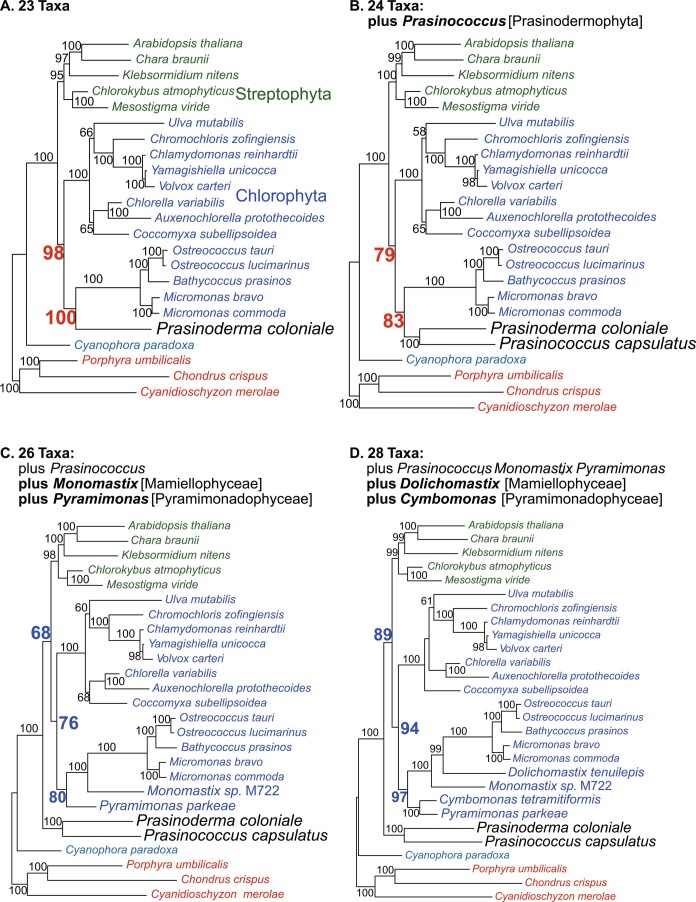
The impact of a severely reduced taxon sampling in rRNA phylogenies on the placement of the Prasinodermophyta. (**a**). RAxML phylogeny of 23 Archaeplastida/Plantae, for which genome sequences have been determined. As an exception, the *Gonium* genome project did not cover both rRNA operons, and thus, Gonium was replaced by the closely related *Yamagishiella*. For similar reasons, *Micromonas pusilla* was replaced by *M. bravo*. The Prasinodermophyta, represented only by *Prasinoderma coloniale*, was resolved as sister to the Mamiellales (Mamiellophyceae) with maximal support. This artificial placement (that is *Prasinoderma coloniale* diverging within the Chlorophyta) gained high support by bootstrapping (numbers in red color). (**b**). Splitting the long branch of *Prasinoderma coloniale* by addition of *Prasinococcus capsulatus* did not change the artificial placement of the Prasinodermophyta, but reduced the bootstrap support for the artificial branches (numbers in red color). (**c**). When the long branch of the Mamiellales was subdivided by addition of *Monomastix* sp. and *Pyramimonas parkeae*, the Mamiellophyceae/Pyramimonadophyceae-clade diverged independently, and the Prasinodermophyta attained a basally diverging position within Viridiplantae. However, the support for the basal divergence of the Prasinodermophyta was relatively low (numbers in blue color). (**d**). Further addition of only two taxa, *Dolichomastix tenuilepis* and *Cymbomonas tetramitiformis*, was sufficient to raise the bootstrap support for the monophyly of the Chlorophyta (to the exclusion of Prasinodermophyta; 94%), and the monophyly of Chlorophyta+Streptophyta (again to the exclusion of Prasinodermophyta; 89%) to high values (numbers in blue color), comparable to the 109-taxa rRNA phylogeny (Fig. 1c), and the genome/transcriptome tree (Fig. 1b). Taxon sampling for resolving the phylogenetic position of the Prasinodermophyta is thus saturated with only 28 sequences of Archaeplastidae/Plantae.

Phylogenetic analyses of *P. coloniale* were performed with two different taxon and datasets. (1) Both maximum-likelihood and Bayesian trees were constructed from an alignment of 256 orthologues of single-copy nuclear genes from 28 taxa of Archaeplastida, showing that *P. coloniale* (and the related *Prasinococcus capsulatus*) diverged before the split of Streptophyta and Chlorophyta (Fig. 1b). All internal branches in the tree received maximal/nearly maximal support, and the monophyly of Streptophyta + Chlorophyta to the exclusion of *P. coloniale* and *P. capsulatus* received 88% bootstrap and 1.0 posterior probability support. A phylogenetic tree constructed from 31 mitochondrial genes of 19 taxa of Archaeplastida also revealed *P. coloniale* as the earliest divergence in Viridiplantae (Supplementary Fig. 4). (2) We increased the taxon sampling to 109 taxa of Archaeplastida, including six sequences of Palmophyllophyceae^27^ comprising nuclear- and plastid-encoded rRNA operons. The phylogeny corroborated the multi-protein phylogeny because Palmophyllophyceae again diverged before the split of Chlorophyta and Streptophyta (Fig. 1c). Separate phylogenies of nuclear- and plastid-encoded rRNA operons gave congruent results, although support values were generally lower (Supplementary Figs. 5 and 6). The summary coalescent method ASTRAL gave inconclusive results (Supplementary Table 5 and Supplementary Figs. 7 and 8), and taxon sampling was sensitive to long-branch attraction^28^ (Supplementary Fig. 9 and Extended Data Fig. 2). The former tree is corroborated by a recent phylotranscriptomic analysis of 1,090 viridiplant species in which the placement of three Palmophyllophyceae was unstable in ASTRAL trees but resolved as the basal divergence of Viridiplantae in concatenated trees^29^. Previous plastome phylogenies placed Palmophyllophyceae either as the earliest divergence within Chlorophyta, sister to all other Chlorophyta^27^, or in an unresolved position among Chlorophyta^15^. Plastome phylogenies are limited by the dataset (70–80 plastid-encoded genes) but also suffer from introgression of the plastid from one species to another, recombination and gene conversion, as well as differential selective pressures acting on protein-coding plastid genes, which may also introduce biases and lead to incongruent gene and species trees^30–33^. For example, unlike nuclear trees, some studies have failed to recover Ulvophyceae, Trebouxiophyceae and Pedinophyceae as monophyletic groups^27^ or Mesostigmatophyceae within Streptophyta^15^. Based on their phylogenetic positions (Fig. 1b,c, Supplementary Figs. 4–6 and Extended Data Fig. 2), gene family comparisons and molecular synapomorphies, we here propose a new division/phylum for the Palmophyllophyceae sensu^27^, the Prasinodermophyta div. nov. with two classes, Prasinodermophyceae class. nov. and Palmophyllophyceae emend (Supplementary Data 1).

### Comparison of gene families among Archaeplastida

**Fig. 2:**
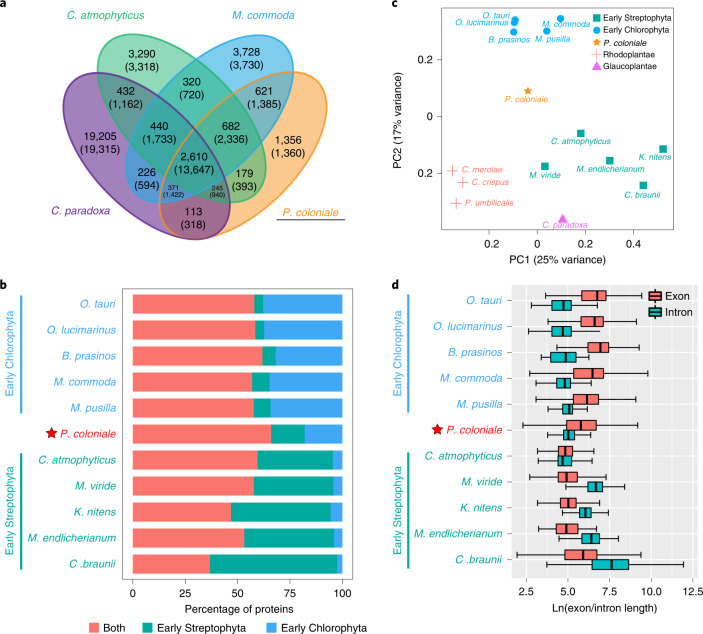
Comparative analysis of *P. coloniale* and other Chlorophyta. **a**, Venn diagram showing unique and shared orthogroups among *P. coloniale*, *C. atmophyticus*, *M. commoda* and *C. paradoxa*. Gene numbers are given in parentheses. **b**, Percentages of proteins found among Viridiplantae (red), Chlorophyta-specific (blue) and Streptophyta-specific (green) based on the classification given in OrthoFinder. Species abbreviations are listed in Supplementary Table 32. **c**, PCA of the type and number of Pfam domains of all genes across the Viridiplantae. **d**, Box-and-whisker plots depicting distributions of the lengths of exons and introns in selected Viridiplantae.

The phylogenetic placement of Prasinodermophyta as a sister group to all other Viridiplantae provided a unique opportunity to reconstruct the minimum core genome of Viridiplantae, and to compare the genome of *P. coloniale* to those of early-diverging Streptophyta, Chlorophyta and the Glaucoplantae, to identify plesiomorphic and apomorphic traits. In total, 4,052 orthogroups are shared among Chlorophyta and Streptophyta, of which 3,292 are also shared with *P. coloniale*. If the orthogroups shared uniquely by *P. coloniale* with either *Micromonas commoda* (621) or *Chlorokybus atmophyticus* (179) are added, 4,092 orthogroups represent the minimal core genome of Viridiplantae (Fig. 2a). A total of 1,356 unique orthogroups were found in *P. coloniale*, mainly involved in biological process categories such as photosynthesis-antenna proteins, plant–pathogen interaction and plant hormone signal transduction (Supplementary Table 6). Thus, it is reasonable to expect that these unique biological traits reflect adaptations of *P. coloniale* to its deep-water/low-light, oligotrophic habitat.

### Comparative genomics of *P. coloniale* with early-diverging Viridiplantae

About 38.5% of the *P. coloniale* genes gave best hits with Chlorophyta, while a similar percentage (33.9%) gave best hits with Streptophyta, supporting an equidistant relationship between *P. coloniale* and Streptophyta and Chlorophyta (Supplementary Fig. 10). *P. coloniale*, along with some representative early-diverging Viridiplantae, showed a very similar percentage of Viridiplantae genes (commonly shared). The remaining proteins of *P. coloniale* were equally distributed among Streptophyta- and Chlorophyta-specific genes (Fig. 2b). Principal component analysis (PCA) showed that early-diverging Chlorophyta (Mamiellophyceae), streptophyte algae (Mesostigmatophyceae, Klebsormidiophyceae and Charophyceae), Glaucoplantae and Rhodoplantae form four separate clusters with *P. coloniale* in an isolated position, which also further supports its classification as a new and independent clade—that is, the Prasinodermophyta div. nov. (Fig. 2c)

**Extended Data Fig. 3:**
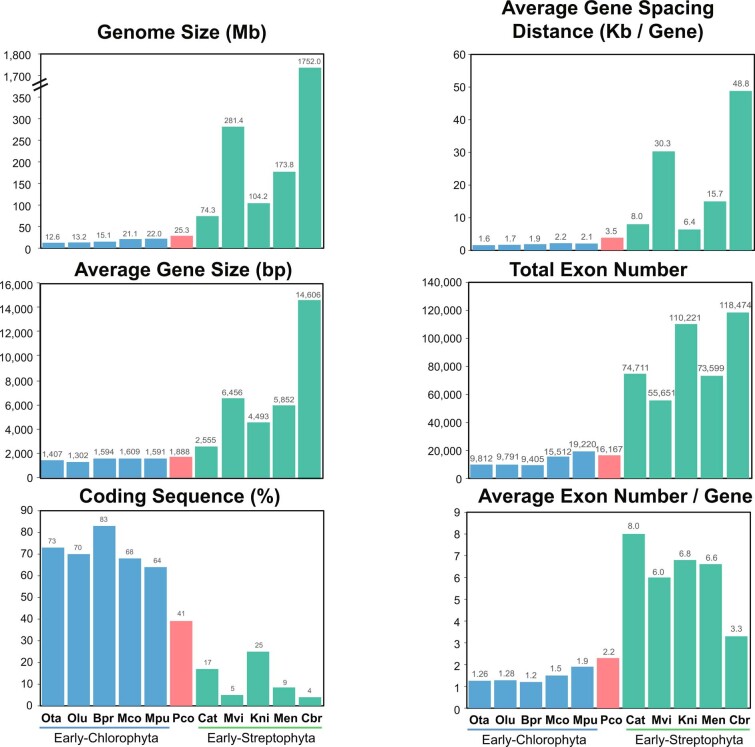
Comparison of genome characteristics across Viridiplantae. Genome size, average gene size, the percentage of the coding sequence, average gene density, average exon number per gene and total exon number among early-diverging lineages of Chlorophyta and Streptophyta compared to *P. coloniale*.

Furthermore, a comparative analysis on structural genomic features showed a trend of gradually increasing average intron length and decreasing average exon length from *P. coloniale* to early-diverging streptophytes, and the opposite trend from *P. coloniale* to early-diverging Chlorophyta was observed (Fig. 2d). In addition, the genome size, gene size, gene spacing distance and total and average exon numbers exhibited a similar pattern with early-diverging Chlorophyta (Extended Data Fig. 3 and Supplementary Table 7). However, the *P. coloniale* genome contains 41% coding sequences, higher than the early-diverging streptophytes but considerably lower than early-diverging Chlorophyta. In summary, the structural characteristics of the *P. coloniale* genome revealed its intermediate position between the extremely compact and small genomes of picoplanktonic early-diverging Chlorophyta^34^ and the larger and structurally more complex genomes of early-diverging streptophytes^35,36^.

### Analysis of transcription factors in *P. coloniale*

In total, 55 of 114 types of TF/TR genes were identified in the *P. coloniale* genome (Supplementary Table 8). Although all 55 types of transcription factor/transcription regulator (TF/TR) genes of *P. coloniale* were also found in Chlorophyta and early-diverging streptophyte algae, considerably lower numbers of TF/TR genes (201) were identified in *P. coloniale* compared to Chlorophyta and Streptophyta (Supplementary Table 9).

**Extended Data Fig. 4:**
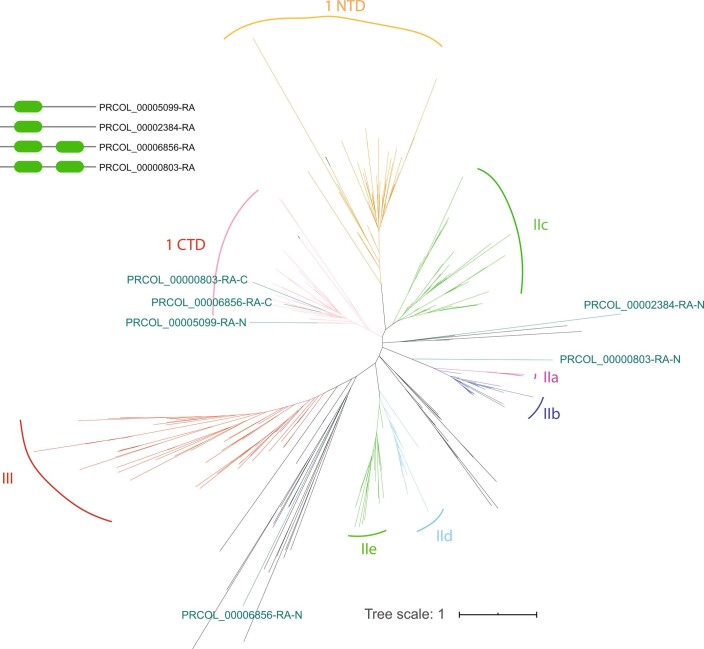
The phylogenetic tree of WRKY domain. *Prasinoderma*’s WRKY domain is marked in light green color. WRKY domains I CTD and I NTD represent the C- and N-terminal domains of a single WRKY gene, each domain is monophyletic comprising both Streptophyta and Chlorophyta. This suggests that the common ancestor of Chlorophyta and Streptophyta had this configuration. Interestingly, *P. coloniale* has four gene copies with a total of six WRKY domains (Supplementary Fig. 13). Two of the gene copies display both N- and C-terminal WRKY domains, the other two have only N-terminal WRKY domains. The phylogenetic tree (Supplementary Fig. 13) placed three WRKY domains in clade I CTD (two C-terminal and one N-terminal WRKY domain), the other N-terminal WRKY domains of *P. coloniale* could not be positioned in one of the 8 WRKY domain subfamilies. We suggest that the I CTD subfamily is ancestral in the Viridiplantae and the N-terminal WRKY domains originated by domain duplication and shuffling.

Among the 55 types of TF/TR genes, the majority (50) are also present in Glaucoplantae and/or Rhodoplantae, suggesting that these constituted the basic TF/TR toolbox in the common ancestor of Archaeplastida. However, five TF/TR types of *P. coloniale* (C2C2-Dof, WRKY, SBP, GARP_ARR-B and TAZ) were presumably gained in the common ancestor of the Viridiplantae since they are absent in both Glaucoplantae and Rhodoplantae (Supplementary Table 8). WRKY proteins are key regulators of development, carbohydrate synthesis, senescence and responses to biotic and abiotic stresses in embryophytes^37^. Using newly retrieved WRKY sequences, we confirmed the presence of eight well-supported WRKY domain subgroups in Viridiplantae (Extended Data Fig. 4). The number of gene copies with WRKY domains and the divergent sequences of the N-terminal WRKY domains in *P. coloniale* may be related to its picoplanktonic lifestyle and/or low-light environment (the picoplanktonic Mamiellophyceae generally also display more than one WRKY gene copy; Supplementary Table 8).

The type-B phospho-accepting response regulator (GARP_ARR-B) family modulates plastid biogenesis, circadian clock oscillation, cytokinin signalling and control of the phosphate starvation response in plants^38^. Since many genes of the cytokinin biosynthesis and signalling pathways are lacking in *P. coloniale* (Supplementary Table 10), these response regulators may be involved in other functions. Finally, the evolution of the SQUAMOSA promotor-binding protein (SBP)-box TF was previously suggested to predate the split of Streptophyta and Chlorophyta^39^. SBP-box TFs have diverse specialized functions in embryophytes, but in green algae they may be involved in more basic functions such as regulation of trace metal homeostasis^40^. The C2C2-Dof (DNA binding with one finger) TFs have been implicated in light control of zygote germination in *Chlamydomonas reinhardtii*^41^ and apparently originated also in the common ancestor of Viridiplantae. Besides the five TF/TR gains, expansion in gene copy numbers was observed in only one TF (Jumonji_Other) in the common ancestor of Viridiplantae when compared with Glaucoplantae and Rhodoplantae. Plant JmjC domain-containing proteins have important functions in both histone modification^42^ and regulation of development and environmental responses^43^.

In contrast to gains and expansion of TFs/TRs, *P. coloniale* also exhibited loss of six TFs/TRs (C2H2, C3H, CCAAT_HAP2, MADS_MIKC, MBF1 and Zinc Finger MIZ type) that are present in Chlorophyta, Streptophyta, Glaucoplantae and Rhodoplantae. Mapping of TFs/TRs on the phylogeny (Fig. 1b) also allowed tentative conclusions about gains of TFs/TRs in the common ancestor of Chlorophyta + Streptophyta (five: ABI3/VP1, Dicer, HD_DDT, Pseudo ARR-B and Whirly) and the common ancestor of Streptophyta (seven: HD-ZIP_I_II, HD-ZIP_III, HD-PLINC, GRF, LUG, SRS and Trihelix).

### Light-harvesting complex (LHC) and LHC-like proteins in *P. coloniale*

Archaeplastida produce metabolic energy by collecting solar energy and transferring it to photosynthetic reaction centres, facilitated by two types of light-harvesting complexes (LHC I and LHC II), composed of LHC proteins that interact with light-harvesting pigments^44–47^. We identified 41 LHC and LHC-like proteins of *P. coloniale* (Supplementary Table 11).

**Fig. 3:**
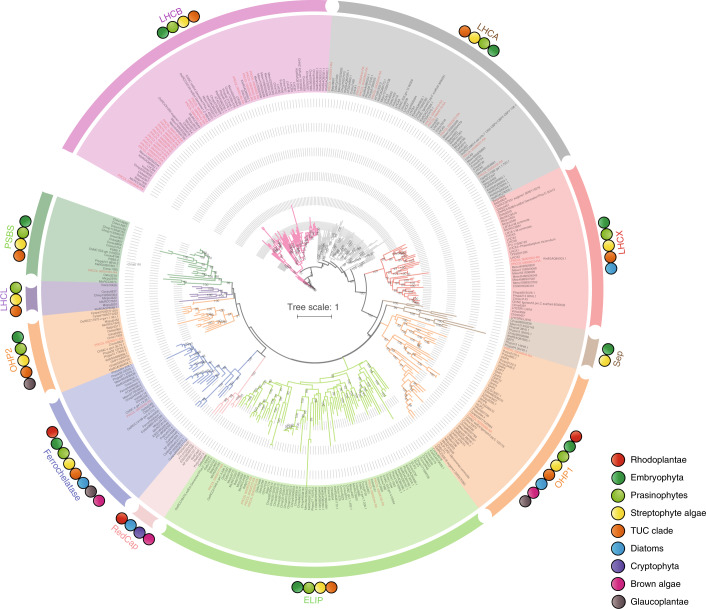
Phylogenetic tree of the LHC antenna protein superfamily. The tree is derived from a MAFFT alignment and was constructed using IQ-TREE (see Methods) with the model of sequence evolution suggested by the programme. Bootstrap values (500 replicates) ≥50% are shown. The LHC superfamily can be divided into ten clades, marked by different colours; the LHC genes of *P. coloniale* are highlighted in red. The coloured circles on the outer ring denote the distribution of the different LHC subfamilies in the respective taxa. The TUC clade comprises Trebouxiophyceae, Ulvophyceae and Chlorophyceae (all in Chlorophyta). ELIP, early light-induced protein; SEP, two-helix stress-enhanced protein; OHP, one-helix protein; PSBS, the photosystem II subunit S; RedCAP, red lineage chlorophyll a/b-binding (CAB)-like protein.

Phylogenetic analysis of LHC proteins from *P. coloniale* showed them to be widely distributed in seven of the ten LHC clades, namely LHCA, LHCB, LHCX, PSBS, OHP, Ferrochelatase and ELIPs (Fig. 3). The *P. coloniale* genome has 19 *Lhcb* genes (six of which apparently originated from three successive gene duplications in the *Prasinoderma* lineage). *P. coloniale* also displayed nine *Lhca* genes, whereas in most of the investigated early-diverging Chlorophyta/Mamiellophyceae and in *Mesostigma* (Streptophyta) there are only six *Lhca* genes (Supplementary Table 11). As in the early-diverging Mamiellophyceae, *P. coloniale* displayed two LHCX proteins. There are three helix proteins in *P. coloniale*, as in *Cyanophora paradoxa*. Other types of LHC-like proteins, such as RedCap, SEP (SEP apparently originated in streptophyte algae) and LHCL, are missing in *P. coloniale*. The relatively large number of gene copies of chlorophyll-a/b-binding proteins (*Lhca*, *Lhcb)* in *P. coloniale* could reflect adaptation to the low-light environment from which this strain was isolated (150 m depth), requiring larger LHC antennae. This is corroborated by two other observations: first, the relatively low Chl-a/b ratio (1.13) reported for this organism and the related *Palmophyllum*^48^ (0.64), and second, the lower number (six) of ELIPs in *P. coloniale* compared to core Chlorophyta (nine in *Ulva lactuca* and ten in *C. reinhardtii)* or early-diverging subaerial/terrestrial streptophyte algae (13/9 in *C. atmophyticus* and *Klebsormidium nitens*, respectively).

### Carbon-concentrating mechanisms (CCMs)

**Fig. 4:**
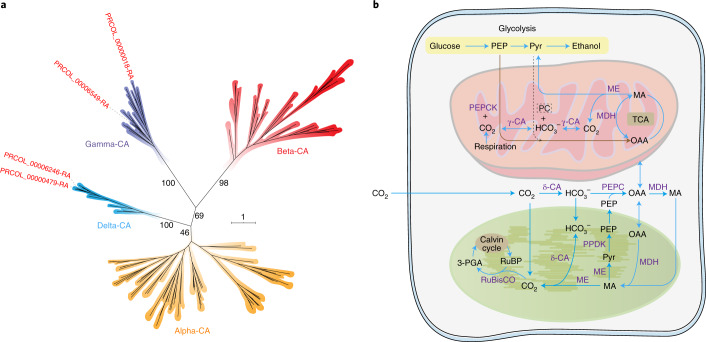
CCMs in *P. coloniale*. **a**, Maximum-likelihood phylogeny of CA proteins in *P. coloniale*. **b**, Proposed CCMs in which inorganic carbon is assimilated by *P. coloniale* based on predicted protein localizations. A brown arrow denotes that a reaction occurs only in *P. coloniale*, and a grey dotted arrow denotes a reaction that exists in Mamiellophyceae. MA, malic acid; MDH, malate dehydrogenase; ME, malic enzyme; Pyr, pyruvate; 3-PGA, 3-phosphoglyceric acid; PPDK, pyruvate, phosphate dikinase; RuBisCO, ribulose-1,5-bisphosphate carboxylase oxygenase; TCA, tricarboxylic acid cycle.

Previous studies of picoplanktonic Mamiellophyceae suggested that these algae might possess a C_4_-like carbon fixation pathway to alleviate low CO_2_ affinity^49^. A C_4_-like CCM has also been reported in photosynthetic stramenopiles^50–53^. CCMs mainly rely on carbonic anhydrases (CAs) that catalyse the reversible conversion of carbon dioxide to bicarbonate. Four CA genes belonging to the delta- and gamma-type CAs were identified in the genome of *P. coloniale*, while alpha- and beta-type CAs were absent (Fig. 4a). Among Viridiplantae, only Mamiellophyceae were found to encode delta-type CAs (Supplementary Table 12). Whereas alpha-, beta- and gamma-type CAs apparently evolved in the common ancestor of Archaeplastida (alpha- and beta-type CAs were lost in *P. coloniale*, and alpha-type CAs in the later-diverging Mamiellophyceae, perhaps related to cell miniaturization in both groups), delta-type CAs apparently evolved in the common ancestor of Viridiplantae and were independently lost in the core Chlorophyta and in Streptophyta.

Here we propose a putative model of CCMs in *P. coloniale*, based on the targets of the genes that are necessary for inorganic carbon assimilation (Fig. 4b). As a potential C_4_-like CCM, malate dehydrogenase catalyses the reaction to yield malate in the cytosol, mitochondrion and chloroplast (Fig. 4b). Malic enzymes could be transported into the mitochondrion and chloroplast, where they release CO_2_. Previous studies of CCMs in *Micromonas* and *Ostreococcus* suggested that these algae might perform cytosol- and chloroplast-based C_4_-like CCMs^49^. *P. coloniale*, however, potentially harbours cytosol-, chloroplast- and mitochondrion-based CCMs to enhance the ability to concentrate CO_2_ in a low-CO_2_ environment. Interestingly, the *P. coloniale* genome encoded phosphoenolpyruvate carboxykinase (PEPCK) but not pyruvate carboxylase (PC), opposite to the situation in the genomes of Mamiellophyceae^49,54–56^. This result suggests that a distinct CCM might exist in *P. coloniale* that uses phosphoenolpyruvate (PEP) as a substrate from the glycolytic pathway to produce oxaloacetate (OAA) by PEPCK, instead of PC as in the Mamiellophyceae (Supplementary Table 12 and 13).

### Analysis of carbohydrate-active enzymes (CAZymes) and peptidoglycan biosynthesis

The *P. coloniale* genome encoded 34 glycoside hydrolases (GHs) and 83 glycosyltransferases (GTs) belonging to 16 GH and 33 GT families (Supplementary Table 14). The total number of CAZymes was lower than in the early-diverging Chlorophyta and Streptophyta, and even lower than in *Ostreococcus* spp., the smallest eukaryotes (Supplementary Table 15), which probably reflects the simple chemical structure of the *P. coloniale* cell wall (cells are enclosed within thick cell walls^57^). *P. coloniale*, however, harbours all genes involved in the biosynthesis and metabolism of starch (Supplementary Table 16 and Supplementary Fig. 11). Surprisingly, we could not find any enzymes involved in the synthesis or remodelling of the major components of the primary cell wall in embryophytes, such as enzymes of cellulose, mannan, xyloglucan and xylan biosynthesis and degradation. *Chlorella* spp. have been reported to contain a cell wall with components of glucosamine polymers such as chitin and chitosan^58^. However, very few chitosan-related genes were identified in the genome of *P. coloniale* (Supplementary Table 17). Interestingly, some but not many bacteria/archaea-specific protein glycosylation genes could be detected in the *P. coloniale* genome, such as low-salt glycan biosynthesis protein Agl12, low-salt glycan biosynthesis reductase Agl14 and the GT AglE, which are involved in S-layer and cell surface structure biogenesis in bacteria and archaea^59^. Furthermore, seven copies of regulatory response/sensor proteins, homologous to bacteria, could be identified in *P. coloniale*, which might respond to environmental signals. Further studies are needed to biochemically explore the main components of the cell wall of *P. coloniale*.

**Fig. 5:**
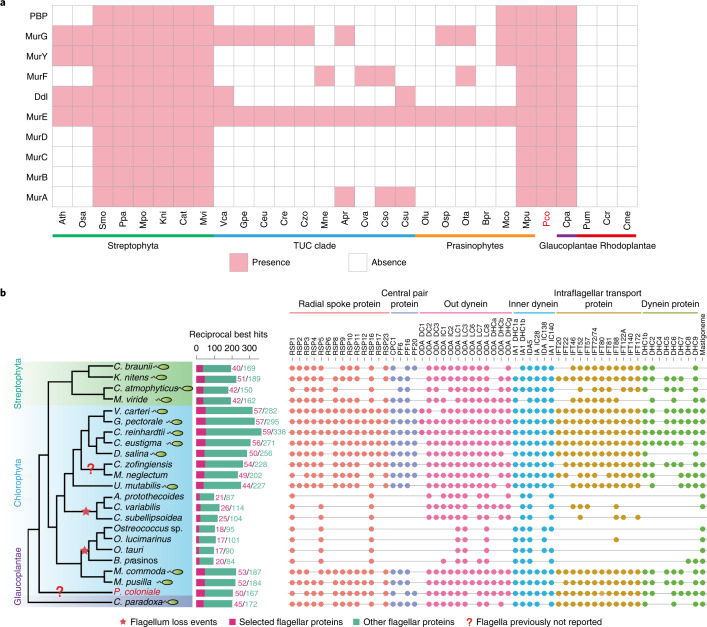
Analysis of peptidoglycan biosynthesis and flagellar proteins derived from the *P. coloniale* genome. **a**, Distribution of proteins involved in the peptidoglycan biosynthetic pathway across Archaeplastida. **b**, Distribution of key flagellar proteins across Viridiplantae and Glaucoplantae.

Peptidoglycan is the main component of cell walls in bacteria^60^. Peptidoglycan biosynthesis requires several enzymes to participate in the conversion of UDP-*N*-acetyl-d-glucosamine (GlcNAc) to GlcNac-*N*-acetylmuramyl-pentapeptide-pyrophosphoryl-undecaprenol^61^. All ten core enzymes involved in peptidoglycan biosynthesis were identified in the *P. coloniale* genome (Fig. 5a). Consistent with previous results, Glaucoplantae (*C. paradoxa*) and *Micromonas pusilla* (Mamiellophyceae), as well as all streptophyte algae, bryophytes and ferns, encoded all the core enzymes^62^. We conclude that peptidoglycan was present in the ancestor of Archaeplastida, completely lost in Rhodoplantae but retained in the common ancestor of Viridiplantae and Glaucoplantae, and then independently lost (to different degrees) in the later-diverging Mamiellophyceae, the core Chlorophyta and the vast majority of vascular seed plants.

### Evolutionary analysis of flagella and sexual reproduction in *P. coloniale*

*Prasinoderma coloniale* and other members of the recently described class Palmophyllophyceae^27^ have been reported to lack flagella^57,63–65^. We performed a comparative analysis of flagellar proteins, and found that non-flagellate species (three species of Trebouxiophyceae and four of *Ostreococcus* and *Bathycoccus*) have ≤26 core flagellar proteins and a total number of flagellar proteins of ≤140 (average 117, *n* = 7), whereas flagellate species display ≥40 core flagellar proteins and a total of ≥192 flagellar proteins (average 272, *n* = 13) (Fig. 5b and Supplementary Table 18). This is corroborated by recent analyses among non-flagellate organisms (angiosperms, Rhodoplantae and pennate diatoms) that yielded on average 77 flagellar proteins^21^ (*n* = 10). Furthermore, non-flagellate species completely lack central pair proteins, dynein heavy chains (DHC1–7), and most of the intraflagellar transport (IFT) and radial spoke proteins (RSP)^21^. RSP3, which binds to the inner dynein arm of the axonemal doublet microtubule and is required for axonemal sliding and flagellar motility, is also absent in non-flagellate organisms^66,67^. The number of flagellar proteins in *P. coloniale* (50 core proteins, 217 total flagellar proteins) and the presence of IFT (12) and DHC proteins (6), as well as RSP3, strongly suggest that *P. coloniale* can produce flagellate cells. The absence of the PF6 protein of the central pair microtubule apparatus may indicate that flagellate cells in *P. coloniale* are short-lived, like the spermatozoids of centric diatoms^68^ or *Chara braunii* that also lack this protein.

Since sexual reproduction has not been observed in *P. coloniale* and Palmophyllophyceae in general, we searched for genes participating in sexual reproduction. Thirty-one out of 40 meiosis-related genes were identified in the *P. coloniale* genome, and 8 out of 11 meiosis-specific genes were found (Supplementary Table 19). These numbers are higher than reported for meiosis-related genes in *Symbiodinium*^69^ (25) and *Trichomonas vaginalis*^70^ (27), and for meiosis-specific genes in some other protists (five genes in *Giardia*^71^ and diatoms^72^ and four in the trebouxiophytes *Auxenochlorella* and *Helicosporidium*^73^), but similar to the number of meiosis-specific genes in *Micromonas* (7)^49^. Interestingly, *P. coloniale* seems to lack DMC1, the loss of which correlates with the adaptation of recombination-independent mechanisms for pairing and synapsis in both *Drosophila* and *Caenorhabditis*^74^. We tentatively conclude that *P. coloniale* retains the capacity for meiotic recombination and thus sexual reproduction.

### De novo NAD^+^ and quinolate biosynthesis in *P. coloniale*

**Fig. 6:**
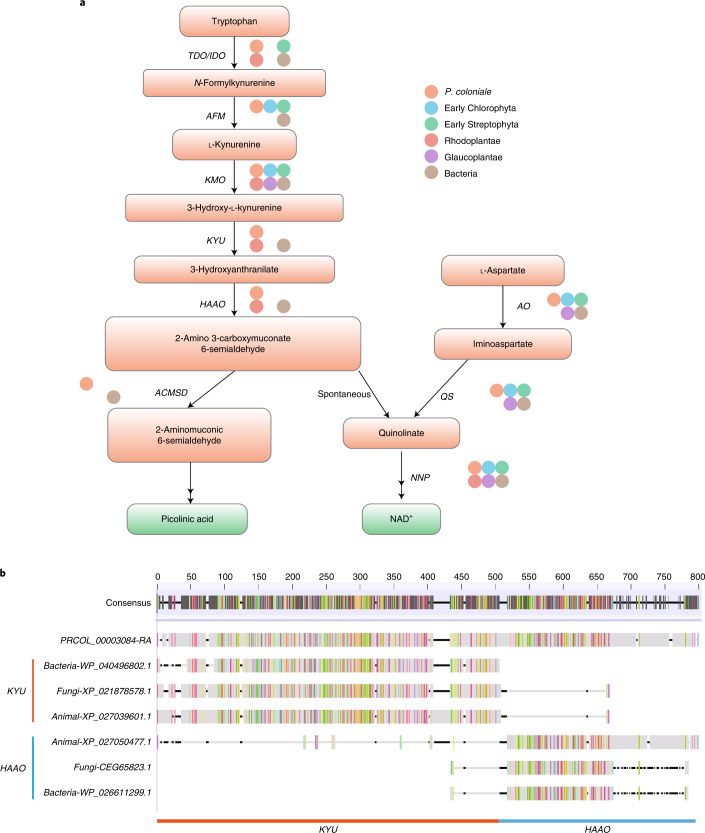
Comparison of de novo NAD^+^ and quinolinate biosynthesis genes. **a**, Distribution of genes related to the de novo NAD^+^ and quinolinate biosynthetic pathways in *P. coloniale* (orange) as compared with Rhodoplantae (red), Glaucoplantae (purple), early-diverging Chlorophyta (blue), early-diverging Streptophyta (green) and bacteria (brown). Solid circles denote the presence of homologues in each clade. TDO/IDO, tryptophan-/indoleamine 2,3-dioxygenase; AFM, arylformamidase; KMO, kynurenine 3-monooxygenase; KYU, kynureninase; HAAO, 3-hydroxyanthranilate 3,4-dioxygenase; ACMSD, 2-amino-3-carboxymuconate-6-semialdehyde decarboxylase; AO, l-aspartate oxidase; QS, quinolinate synthase. **b**, A gene fusion architecture between *KYU* and *HAAO* of *P. coloniale*; the left and right parts are the *KYU* and *HAAO* genes, respectively. A comparison of sequence similarity of various *KYU* and *HAAO* genes from different organisms is shown.

Nicotinamide adenine dinucleotide (NAD) and its phosphate (NADP) are essential redox co-factors in all living systems. All eukaryotic organisms have the ability to synthesize NAD by one of two de novo pathways, the aspartate pathway^75^ or the kynurenine pathway starting with tryptophan^76^. To date, no eukaryotic organism had been found that contains both pathways. *P. coloniale* is the first eukaryotic organism to display both pathways (Supplementary Table 20, Fig. 6a and Supplementary Figs. 12–21): the (presumably) ancestral eukaryotic kynurenine pathway and the aspartate pathway. It has been hypothesized that the latter was acquired through primary endosymbiosis from cyanobacteria^77^. This is corroborated by the fact that both aspartate oxidase (AO) and quinolinate synthase (QS) of Glaucoplantae in phylogenetic analyses branch within cyanobacteria. Rhodoplantae have apparently lost the aspartate pathway (and instead retained the kynurenine pathway for NAD biosynthesis^77^). In Viridiplantae, however, the original cyanobacterial AO and QS genes were replaced by those acquired through horizontal gene transfer (HGT) from other bacteria (Bacteriodetes and Deltaproteobacteria, respectively^77^). However, we can now develop a hypothetical evolutionary scenario for both pathways in Archaeplastida: with the introduction of the aspartate pathway from cyanobacteria during primary endosymbiosis, the ancestral eukaryotic kynurenine pathway for NAD biosynthesis was lost in Glaucoplantae and Viridiplantae (but not in Rhodoplantae, which apparently lost the aspartate pathway). While Glaucoplantae essentially retained the cyanobacterial aspartate pathway, the ancestor of the Viridiplantae replaced the cyanobacterial AO and QS genes by nuclear-encoded genes obtained from other bacteria through HGT, thus compensating their function and representing a new synapomorphy for Viridiplantae. This recalls the situation in *Paulinella chromatophora*, in which loss of genes from the chromatophore genome was compensated by bacterial genes obtained through HGT and encoded on the nuclear genome^78^. We suggest that *P. coloniale* retained the kynurenine pathway, not for NAD synthesis but for synthesis (and possible excretion) of picolinic acid. Additionally, gene fusion architecture between KYU and HAAO of *P. coloniale* was observed (Fig. 6b). The metabolite picolinic acid, a tryptophan catabolite, can potentially form metal complexes with limiting trace elements such as iron, an important property in oligotrophic environments.

### Vitamin auxotrophy and selenocysteine-containing proteins in *P. coloniale*

Previous studies found that some Mamiellophyceae (*Ostreococcus*, *Micromonas*) need to acquire the vitamins thiamine (B_1_) and cobalamin (B_12_) from the extracellular environment for growth, because they lack either enzymes needed for their biosynthesis (B_1_) or vitamin-independent isoforms of essential enzymes (for example, methionine synthase) that require the vitamin as a co-enzyme (B_12_)^79,80^. *P. coloniale* shares these features with the picoplanktonic Mamiellophyceae (Supplementary Table 21). The absence of any enzymes involved in the biosynthesis of thiamine in *P. coloniale* (except for the final phosphorylation step) demonstrates (1) the lack of bacterial contaminations in the genome assembly of the axenic strain used, and (2) that thiamine must be provided by the extracellular environment. It has recently been shown that, in *Ostreococcus tauri*, B_1_ and B_12_ auxotrophy can be alleviated by co-cultivation with the bacterium *Dinoroseobacter shibae*, a member of the Rhodobacteraceae^81^, suggesting that in *P. coloniale* similar algal/bacterial partnerships may exist. Most importantly, *P. coloniale* is also a vitamin B_7_ (biotin) auxotroph, lacking all four genes involved in the biosynthesis of this vitamin, and apparently the only biotin auxotroph currently known among Archaeplastida^82^ (Supplementary Table 21). Many genomes of marine bacteria contain full sets of biotin biosynthesis genes^83,84^ and these bacteria could be a source of biotin for *P. coloniale*. Genome compaction in these picoplanktonic eukaryotes may have facilitated evolution of such symbiotic interactions in an oligotrophic marine environment.

The Mamiellophyceae genomes encode a large number of selenocysteine-containing proteins compared to *C. reinhardtii*^49,54^. The number of selenoproteins, their homologues and selenocysteine insertion sequences have recently been investigated across selected Archaeplastida genomes^85^. *P. coloniale* also displayed a high number of selenocysteine-containing proteins, similar to picoplanktonic Mamiellophyceae but unlike core Chlorophyta, early-diverging streptophyte algae, Glaucoplantae and Rhodoplantae (Supplementary Table 22). Selenoenzymes are more catalytically active than similar enzymes lacking selenium, and small cells may therefore require fewer of those proteins^54^.

## Conclusion

The picoplanktonic eukaryote *P. coloniale* is a member of a new division/phylum of Viridiplantae, the Prasinodermophyta, that in phylogenomic analyses diverges before the split of Viridiplantae into Chlorophyta and Streptophyta. Its genome revealed both ancestral and derived characteristics that correspond to its unique phylogenetic position, equidistant from Chlorophyta and Streptophyta. The genome of strain CCMP 1413 showed adaptations to a low-light (deep-water), oligotrophic oceanic environment. In such an environment, metabolic coupling and horizontal gene transfer from bacteria may have facilitated adaptation. In the latter, it resembles the genomes of the picoplanktonic Mamiellophyceae (Chlorophyta), although a number of apomorphic features in the genome of *P. coloniale* suggest that the picoplanktonic lifestyle in the two groups evolved independently.

## Methods

### Cultivation of algae, nucleic acid extraction and light microscopy

Cultures of *P. coloniale* (CCMP 1413) were obtained from the National Center for Marine Algae and Microbiota (https://ncma.bigelow.org/ccmp1413#.XqP0zGgzYdU). Axenic cultures were prepared by streaking out algae on agar and picking single cell-derived clones from the plates. Algae were grown in a modified ASP12 culture medium^86^ (http://www.ccac.uni-koeln.de/) in a 14/10 h light/dark cycle at 20 µmol photons m^−2^ s^−1^ and 23 °C. During all steps of culture scale-up until nucleic acid extraction, axenicity was monitored by both sterility tests and light microscopy. Total RNA was extracted from *P. coloniale* using the CTAB-PVP method as described in ref. ^87^ (appendix S1 therein). Total DNA was extracted using a modified CTAB protocol^88^. Light microscopy was performed with a Leica DMLB light microscope using a PL-APO ×100/1.40 numerical aperture (NA) objective, an immersed condenser (NA 1.4) and a Metz Mecablitz 32 Ct3 flash system.

### Genome sequencing and assembly

The long reads libraries were constructed using standard library preparation protocols and sequenced by the Pacbio Sequel platform. NextDenovo (https://github.com/Nextomics/NextDenovo) was used to generate the draft assembly. The draft assembly were first polished by Pacbio reads using Arrow, then NextPolish was used to perform a second round of polishing using short reads generated by the Illumina sequence platform. To eliminate putative bacterial contamination, contigs were searched against the NCBI non-redundant database.

*K*-mer analysis was performed to survey genome size, heterozygosity and repeat content before genome assembly. The peak of *K*-mer frequency (*M*) was determined by the real sequencing depth of the genome (*N*), read length (*L*) and the length of the *K*-mer (*K*) following the formula: *M* = *N* × (*L* – *K* + 1)/*L*. This formula enables accurate estimation of *N*, and hence an estimation of the genome size for homozygous diploid or haploid genomes. All these analyses indicated homozygosity of the genome and gave similar estimations of genome size. The final genome size was estimated (~26.04 Mb) using 17-mer analysis.

The quality of the assembly was evaluated in four ways: (1) we used BUSCO v.3 to determine the presence of a proportion of a core set of 303 highly conserved eukaryotic genes. (2) SOAP (v.2.21) was used to map the short reads to the assemblies to evaluate the DNA reads mapping rate in both species. In the meantime, sequence depth and genomic copy content distribution were calculated. (3) We used BLAT (v.36) to compare the draft assemblies to a transcript assembled by Bridger. (4) We mapped the RNA reads to the draft assemblies to evaluate the RNA reads mapping rate using Tophat2.

### Transcriptome sequencing and assembly

Two methods of library construction were performed. The rRNA-depleted RNA library was constructed using the ribo-zero rRNA removal kit (plant) (Illumina) following the manufacturer’s protocol, while the poly (A)-selected RNA library was constructed using the ScriptSeq Library Prep kit (Plant leaf) (Illumina) following the manufacturer’s protocol. A total of 12.09 Gb of PE-100 RNA-seq data was generated using the Illumina Hiseq 4000 sequencing platform. SOAPfilter (v.2.2) was used to filter the reads with *N* > 10 bp, removing duplicates and adaptors. As a result, 5.58 Gb of clean reads were obtained after filtering, then Bridger was used to assemble the clean data into a transcriptome, which was used for gene annotation and genome evaluation.

### Repeat annotation

A pipeline combining de novo and library-based approaches was used to identify the repeat elements. For the de novo approach, MITE-hunter and LTRharvest were used to annotate the transposon and retrotransposon, respectively, then RepeatModeler (v.1.0.8) was performed to annotate the other repeat elements. For the library-based approach, the custom library Repbase 22.01 was used to identify the repeat elements by RepeatMasker.

### Gene prediction and preliminary functional annotation

Three methods were combined to predict the gene model, an ab initio prediction method, a homologue search method and a RNA-seq data-aided method. For the first method, PASApipeline-2.1.0 was performed to predict gene structure using transcripts assembled by Bridger, which were further used in AUGUSTUS (v.3.2.3) to train gene models. GeneMark (v.1.0) was used to construct a hidden Markov model (HMM) profile for further annotation. For the homologue search method, gene sets of homologue species and public proteins of *Prasinoderma* were downloaded from the NCBI database. For the RNA-aided method, transcripts were assembled by Bridger as evidence. All predictions were combined using two rounds of MAKER (v.2.31.8) to yield the consensus gene sets. The final gene set was evaluated by mapping with eukaryotic BUSCO v.3 dataset and RNA read mapping by Tophat2. Coverage depth was calculated by Samtools (v.0.1.19).

Preliminary gene function annotation was performed by BLASTP (<10 × 10^–5^) against certain known databases, including SwissProt, TrEMBL, KEGG, COG and NR. InterProScan (using data from Pfam, PRINTS, SMART, ProDom and PROSITE) was used to identify protein motifs and protein domains of the predicted gene set. Gene Ontology information was obtained through Blast2Go (v.2.5.0). For certain key functional genes we used a stricter functional annotation method by the addition of some known query genes, as described in Detection of important candidate functional genes.

### Whole-genome phylogenetic analysis

For whole-genome phylogenetic analysis, both genome data downloaded from public databases (NCBI Refseq or JGI) and transcriptome data downloaded from the 1000 Plants Project (1KP, https://sites.google.com/a/ualberta.ca/onekp/) were used. First, OrthoFinder (v.1.1.8) was used to infer orthogroups (gene families) among the 28 selected organisms. Single-copy orthogroups (gene families with only one gene copy per species) were collected, since every single-copy gene in each gene family could be an orthologue among 28 organisms. We used multiple alignment with fast Fourier transform (MAFFT v.7.310) to perform multiple sequence alignment for each single-copy gene orthogroup, followed by a gap position (removing only positions where 50% or more of the sequences having a gap are treated as gap positions). We constructed multiple phylogenetic trees using different tree construction methods (concatenated and coalescent methods) based on different taxon samplings (that is, number of species). In concatenated tree reconstruction, each single-copy gene alignment was linked by order to establish a super-gene, which was used to construct a concatenated maximum-likelihood phylogenetic tree with either RAxML (amino acid substitution model: CAT + GTR, with 500 bootstrap replicates) or IQ-TREE (amino substitution model inferred by ModelFinder, with 500 bootstrap replicates). In addition, we used MrBayes (v.3.2.6) to construct a Bayesian phylogenetic tree, Markov chain Monte Carlo, which was set to run 1,000,000 generations and sampled every 1,000 generations, the first 25% of which was discarded as burn-in. In the coalescent method, a maximum-likelihood phylogenetic tree was constructed for each single-copy orthogroup. We then used ASTRAL to combine all single-copy gene trees into a species tree with the multi-species coalescent model. Finally, we compared and summarized phylogenetic trees using different methods or different datasets. For a general discussion on concatenated versus coalescent methods for phylogenetic reconstruction, see ref. ^28^.

### Phylogenetic analyses of complete nuclear and plastid-encoded rRNA operon sequences of 109 Archaeplantae

New sequence data of rRNA operons were generated for several taxa (see Supplementary Table 33, and as described previously^30^). For other taxa, data were either retrieved from annotated entries in sequence databases (https://www.ncbi.nlm.nih.gov/nucleotide/) or assembled from non-annotated transcriptome sequence data (MMETSP and ONE_KP; see Supplementary Table 31). All new rRNA sequences, as well as newly assembled transcriptome data, were submitted to GENBANK (https://www.ncbi.nlm.nih.gov/genbank/; bold accession numbers in Supplementary Table 31). Sequences were manually aligned, guided by rRNA/transfer RNA secondary structures using SeaView 4.3.0 (http://pbil.univ-lyon1.fr/software/seaview.html). For phylogenetic analyses, only those positions were selected that could be unambiguously aligned among the Rhodoplantae, Glaucoplantae and Viridiplantae—in total, 8,818 nucleotides (nt). For all phylogenetic analyses, the 8,818 positions were subdivided into four sections: nuclear 18 S rDNA (1,621 nt), nuclear 5.8 S and 28 S rDNA (3,025 nt), plastid 16 S rDNA and two tRNA genes (1,535 nt) and plastid 23 S rDNA (2,637 nt). Tree reconstructions were performed at the CIPRES Science Gateway (http://www.phylo.org/sub_sections/portal/) using three methods: maximum-likelihood with RAxML (v.8.2.10), maximum-likelihood with IQ-TREE (v.1.6.10) and Bayesian tree reconstruction with MrBayes (v.3.2.6).

RAxML analyses were performed with 1,000 bootstrap replicates, each with 100 starting trees, using either the GTRGAMMA model (for all trees shown here) or the GTRCAT model (GTRCAT trees were almost identical to GTRGAMMA trees; not shown). In likelihood analyses using IQ-TREE, the best-fitting model was identified by ModelFinder and the bootstrap analysis again involved 1,000 replicates. For Bayesian analysis, 1,000,000 generations were calculated under the GTR + I + G model, and generations 1–250,000 were discarded as burn-in. Bootstrap percentages <50% and Bayesian posterior probabilities <0.9 were regarded as ‘unsupported’. Phylogenetic trees were also constructed using nuclear- and plastid-encoded rRNA operons separately (Supplementary Figs. 5 and 6).

### Search for unique rRNA synapomorphies

To find unique molecular synapomorphies (Supplementary Files_Taxonomic Acts and Revisions)—that is, rare mutations that characterize a given clade—we performed tree-based synapomorphy searches as previously described^89^. To identify genuine non-homoplasious synapomorphies (flagged as NHS), and to find homoplasious changes (parallelisms and reversals), the synapomorphy search must cover as much diversity as possible. Therefore, all synapomorphies that resulted from the initial search procedure (using only 109 Plantae) were controlled for homoplasies by (1) a taxon-rich alignment containing nuclear rRNA operons from about 1,300 Archaeplastida/Plantae, (2) an alignment with plastid rRNA operons from about 1,600 Archaeplastida/Plantae and (3) BLAST searches (https://blast.ncbi.nlm.nih.gov/Blast.cgi).

### Genome composition of *P. coloniale* genome

We looked at the components of the *P. coloniale* genome mainly in three ways: (1) gene family clustering was first performed on gene sets of the species *C. atmophyticus* (Streptophyta), *M. commoda* (Chlorophyta), *C. paradoxa* (Glaucoplantae) and *P. coloniale*. Commonly shared and unique gene families were shown and displayed on a Venn diagram. (2) The *P. coloniale* gene set was aligned to the NCBI non-redundant database (NR), and the best alignment results (Best-hit) were obtained for each gene. The NCBI taxonomy database was then used to classify the *P. coloniale* gene set. (3) We selected early-diverging Streptophyta (five species), early-diverging Chlorophyta (five species) and *P. coloniale* to perform the gene family cluster, and then divided all gene families into three categories: early Chlorophyta gene families, early Streptophyta gene families and gene families shared by both early Chlorophyta and early Streptophyta^35^. First, we removed unusual/weird gene families in which the gene number of some species was over tenfold larger than the average gene number of the other species. We also removed gene families that include only one species. Then, the average gene numbers in early Chlorophyta and early Streptophyta were determined for each gene family. If the average gene number of early Chlorophyta in a gene family was more than twice the number of early Streptophyta, that gene family was designated as an early Chlorophyta gene family. Conversely, if the average gene number of early Streptophyta in a gene family was larger than twice the number of early Chlorophyta, that gene family was designated as an early Streptophyta gene family. The remaining gene families were shared between early Chlorophyta and early Streptophyta.

### Detection of key candidate functional genes

All candidate genes were screened based on the following conditions: (1) candidate gene sequences should be similar to the query genes collected from previous studies or databases (BLAST <10 × 10^–5^); and (2) the function of the candidate genes should be consistent with the query genes according to online NR functional annotation or Swissprot functional annotation.

Regarding the detection of flagellar genes, we mainly referenced the flagellar genes from refs. ^49,90^. After elimination of redundancy, we obtained 397 flagellar genes as our query set. We used the reciprocal best hits method to identify flagellar genes.

For cell wall-related gene annotation we used the CAZyme database as query, then the web meta-server dbCAN2 (http://bcb.unl.edu/dbCAN2/index.php) was used to detect CAZymes. dbCAN2 integrates three tools/databases for automated CAZyme annotation: (1) HMMER, for annotation of the CAZyme domain against the dbCAN CAZyme domain HMM database; (2) DIAMOND for fast blast hits in the CAZy database; and (3) Hotpep for short conserved motifs in the Peptide Pattern Recognition (PPR) library.

For TFs we used the HMMER search method. We downloaded the HMMER model of the domain structure of each transcription factor from the Pfam website (https://pfam.xfam.org/) while referring to the TAPscan v.2 transcription factor database^91^ (https://plantcode.online.uni-marburg.de/tapscan/). Preliminary candidates were collected by searching the profile HMM for each species (<10 × 10^–5^), then we filtered those genes that did not match the SwissProt functional annotation (<10 × 10^–5^). Finally, we filtered genes containing a wrong domain according to the domain rules of the TAPscan v.2 transcription factor database. Most TFs/TRs were confirmed by phylogenetic tree analysis.

### Subcellular localization

To predict where key proteins (for example, certain enzymes related to carbon-concentrating mechanisms) reside in a cell, we used online tools including WoLF_PSORT (https://www.genscript.com/wolf-psort.html?src=leftbar), TargetP (http://www.cbs.dtu.dk/services/TargetP/), Hectar (https://webtools.sb-roscoff.fr/) and LocSigDB (http://genome.unmc.edu/LocSigDB/index.html) to predict the subcellular localization of these proteins. Combining the results of the four tools, we estimated the localization.

### Reporting Summary

Further information on research design is available in the Nature Research Reporting Summary linked to this article.

## Supplementary information

Supplementary Information Supplementary Figs. 1–21.

Reporting Summary

Supplementary Tables Supplementary Tables 1–33.

Supplementary Data 1 Taxonomic Acts and Revisions.

## Data Availability

Whole-genome assemblies, annotation and raw data for *P. coloniale* in this study are deposited at the CNGB Nucleotide Sequence Archive^92^ (CNSA: http://db.cngb.org/cnsa, accession no. CNP0000924).
