# Supplementary material for: The genome of Prasinoderma coloniale unveils the existence of a third phylum within green plants
Source: Nat Ecol Evol. 2020 Jun 22;4(9):1220–31. doi: 10.1038/s41559-020-1221-7 (PMC7455551; doi:10.1038/s41559-020-1221-7)
Supplement: Supplementary file 1 — Supplementary Figs. 1–21. [file 41559_2020_1221_MOESM1_ESM.pdf]

nature ecology & evolution

SUPPLEMENTARY INFORMATION
https://doi.org/10.1038/s41559-020-1221-7

In the format provided by the authors and unedited.

The genome of *Prasinoderma coloniale* unveils the existence of a third phylum within green plants

Linzhou Li[1,2,13], Sibo Wang[1,3,13], Hongli Wang[1,4], Sunil Kumar Sahu[1], Birger Marin[5], Haoyuan Li[1], Yan Xu[1,4], Hongping Liang[1,4], Zhen Li[6], Shifeng Cheng[1], Tanja Reder[5], Zehra Çebi[5], Sebastian Wittek[5], Morten Petersen[3], Barbara Melkonian[5,7], Hongli Du[8], Huanming Yang[1], Jian Wang[1], Gane Ka-Shu Wong[1,9], Xun Xu[1,10], Xin Liu[1], Yves Van de Peer[6,11,12] ✉, Michael Melkonian[5,7] ✉ and Huan Liu[1,3] ✉

[1]State Key Laboratory of Agricultural Genomics, BGI-Shenzhen, Shenzhen, China. [2]Department of Biotechnology and Biomedicine, Technical University of Denmark, Lyngby, Denmark. [3]Department of Biology, University of Copenhagen, Copenhagen, Denmark. [4]BGI Education Center, University of Chinese Academy of Sciences, Shenzhen, China. [5]Institute for Plant Sciences, Department of Biological Sciences, University of Cologne, Cologne, Germany. [6]Department of Plant Biotechnology and Bioinformatics (Ghent University) and Center for Plant Systems Biology, Ghent, Belgium. [7]Central Collection of Algal Cultures, Faculty of Biology, University of Duisburg-Essen, Essen, Germany. [8]School of Biology and Biological Engineering, South China University of Technology, Guangzhou, China. [9]Department of Biological Sciences and Department of Medicine, University of Alberta, Edmonton, Alberta, Canada. [10]Guangdong Provincial Key Laboratory of Genome Read and Write, BGI-Shenzhen, Shenzhen, China. [11]College of Horticulture, Nanjing Agricultural University, Nanjing, China. [12]Centre for Microbial Ecology and Genomics, Department of Biochemistry, Genetics and Microbiology, University of Pretoria, Pretoria, South Africa. [13]These authors contributed equally: Linzhou Li, Sibo Wang. ✉e-mail: yves.vandepeer@psb.vib-ugent.be; michael.melkonian@uni-koeln.de; liuhuan@genomics.cn

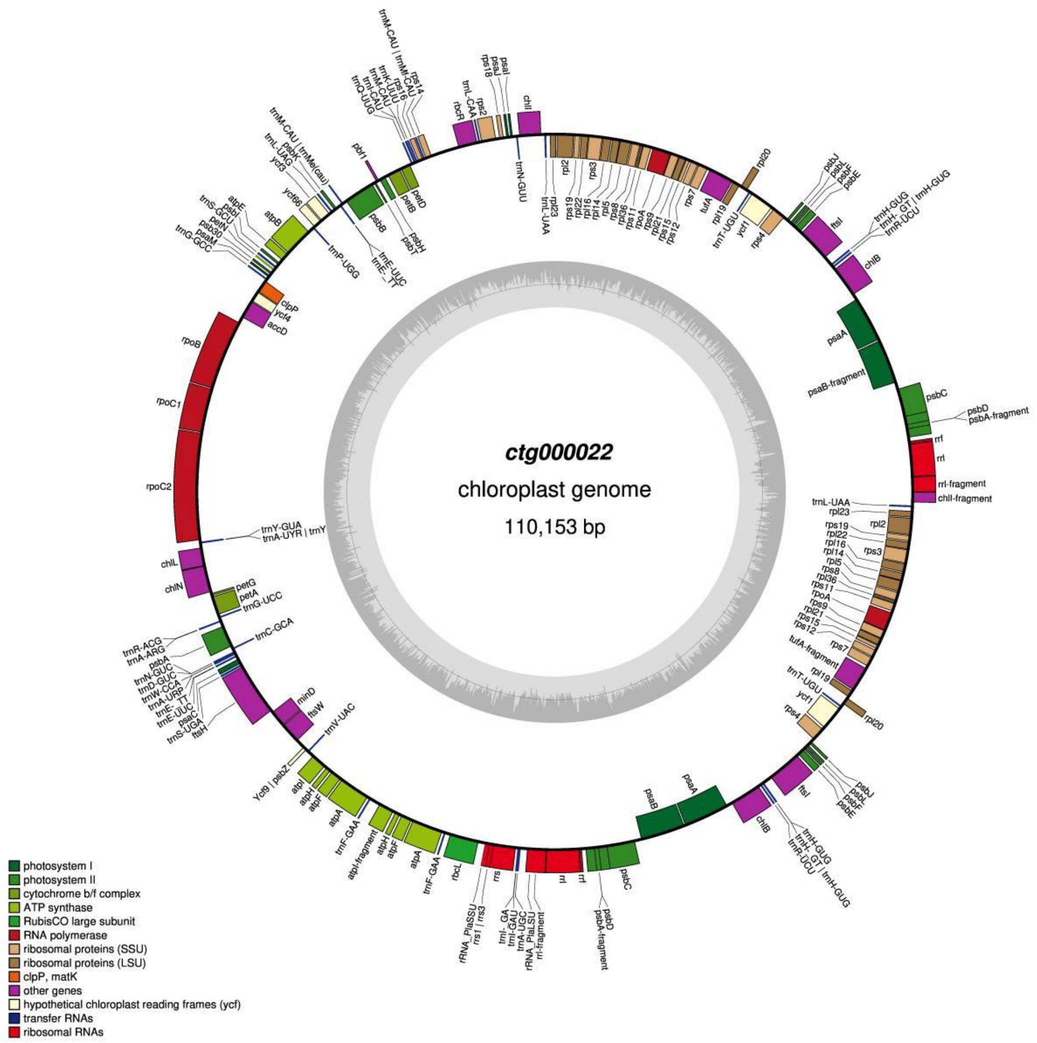

Supplementary Figure 1. The chloroplast circos map of *Prasinoderma coloniale*.

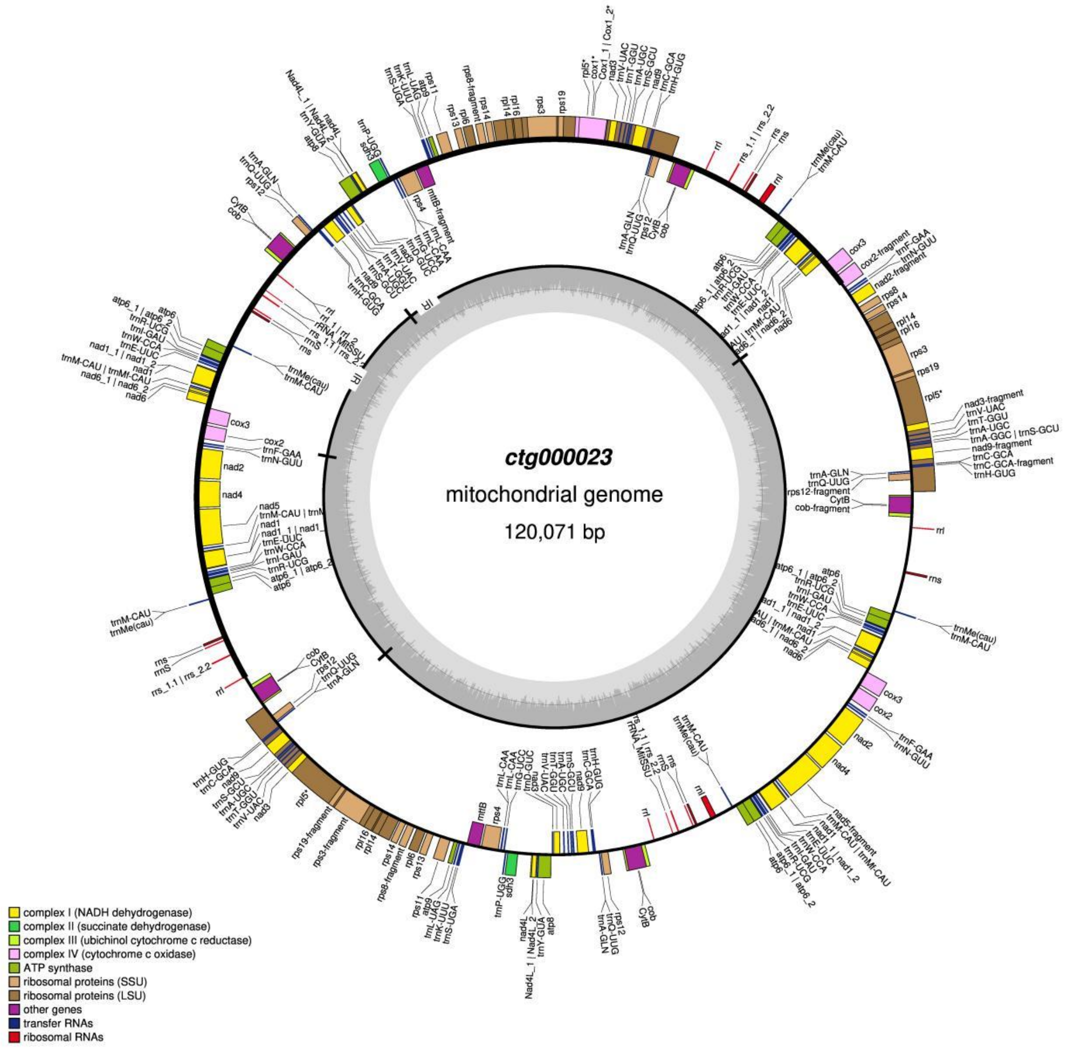

Supplementary Figure 2. The mitochondrial circos map of *Prasinoderma coloniale*.

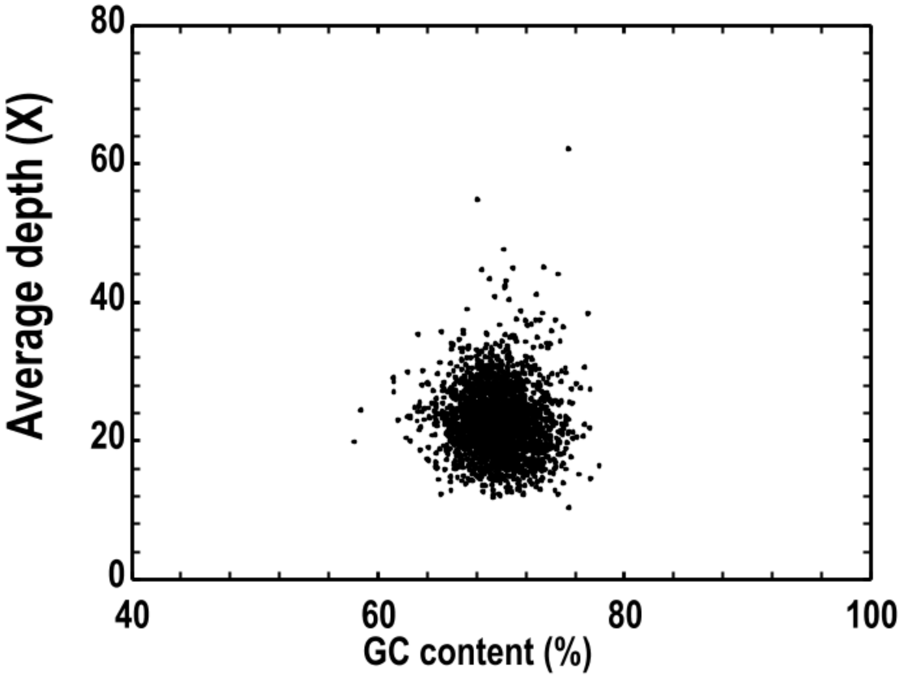

Supplementary Figure 3. The GC depth distribution of *Prasinoderma coloniale*.

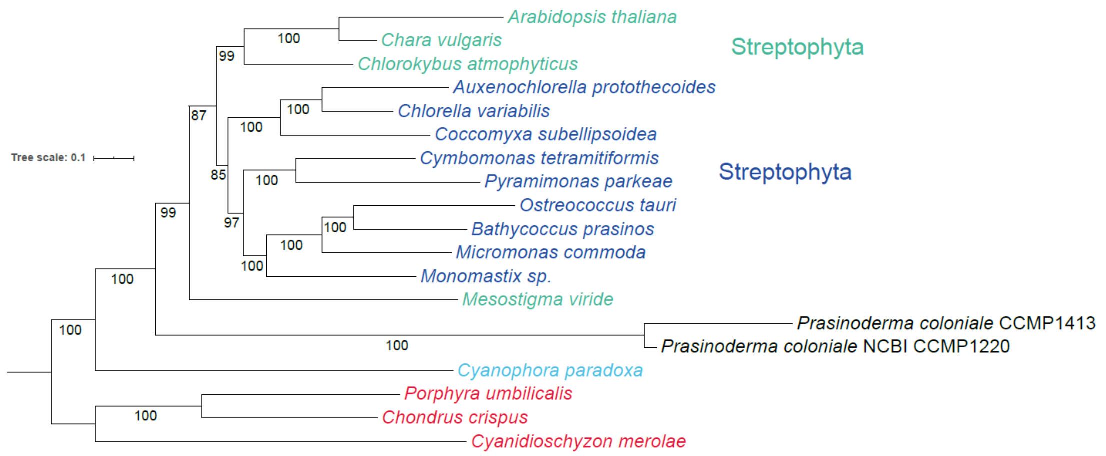

Supplementary Figure 4. The concatenated phylogenetic tree of 19 species with 31 single-copy mitochondria gene place Prasinodermophyta (*Prasinoderma coloniale*) to the sister lineage of other Viridiplantae with high bootstrap support (100.0).

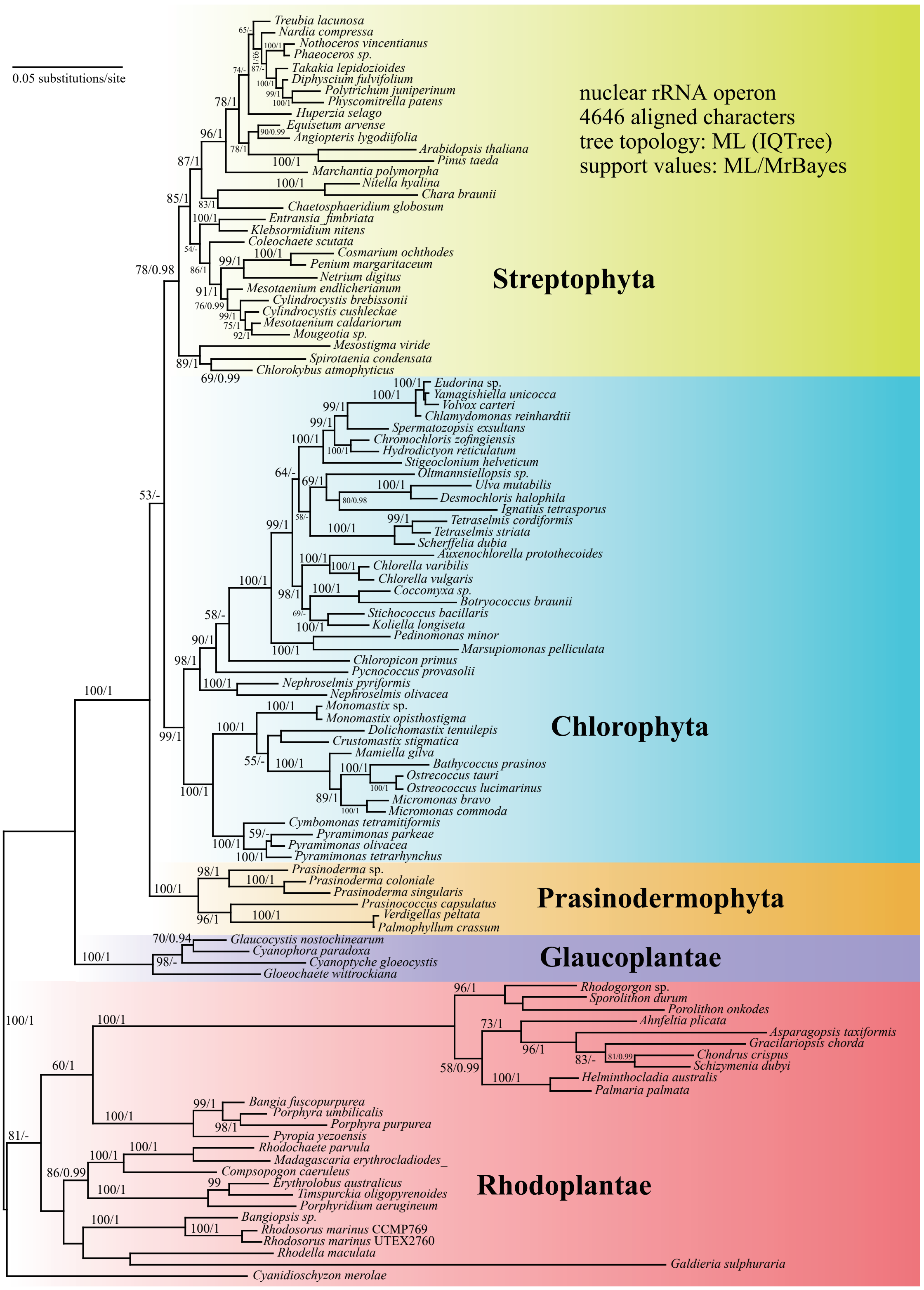

Supplementary Figure 5. The nuclear rRNA operon tree of 109 species with 4,646 aligned characters place Prasinodermophyta (*Prasinoderma coloniale*) to the sister lineage of other Viridiplantae with high bootstrap support (100.0/1).

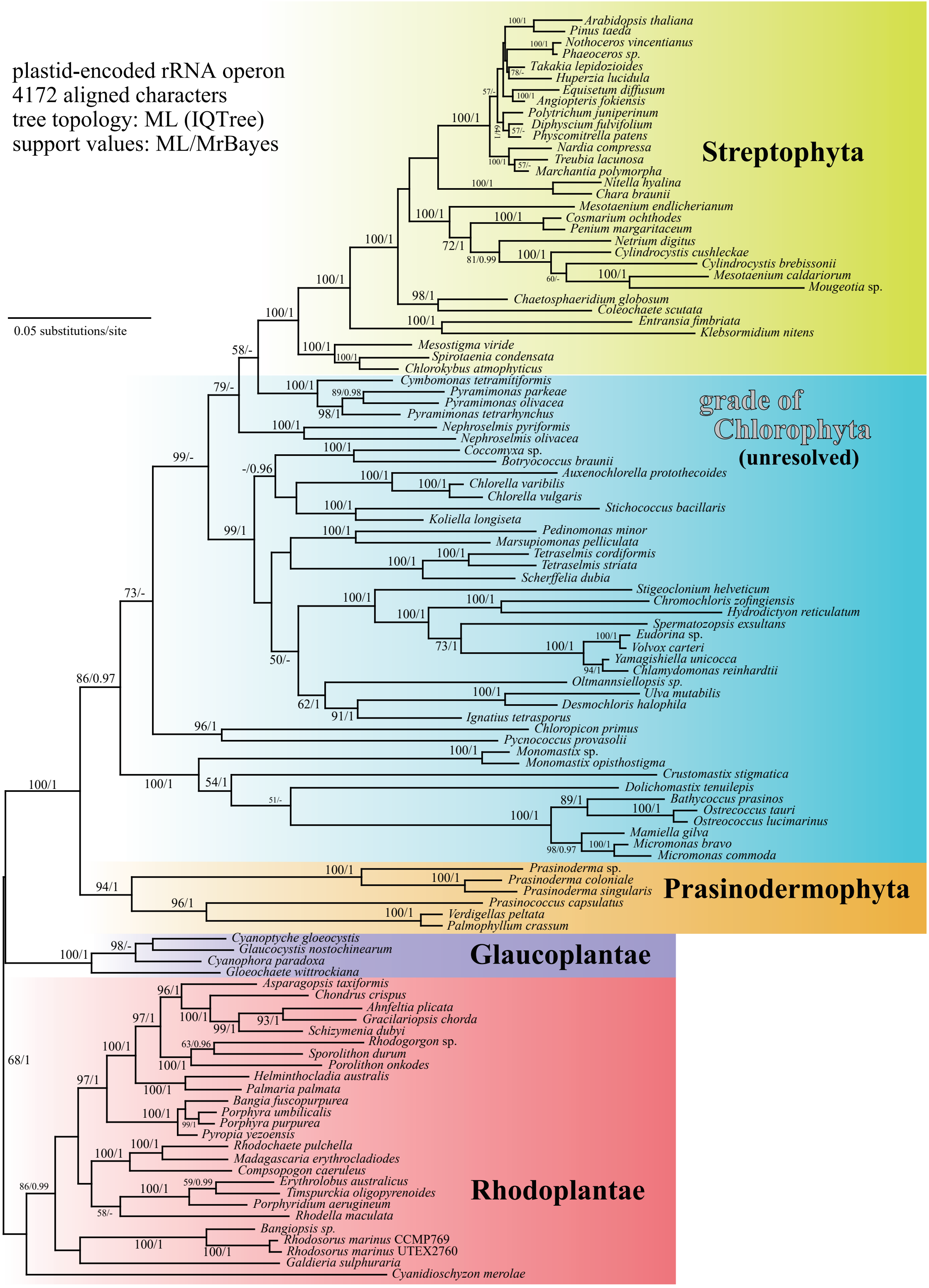

Supplementary Figure 6. The plastid-encoded rRNA operon tree of 109 species with 4,172 aligned characters place Prasinodermophyta (*Prasinoderma coloniale*) to the sister lineage of other Viridiplantae with high bootstrap support (94.0/1).

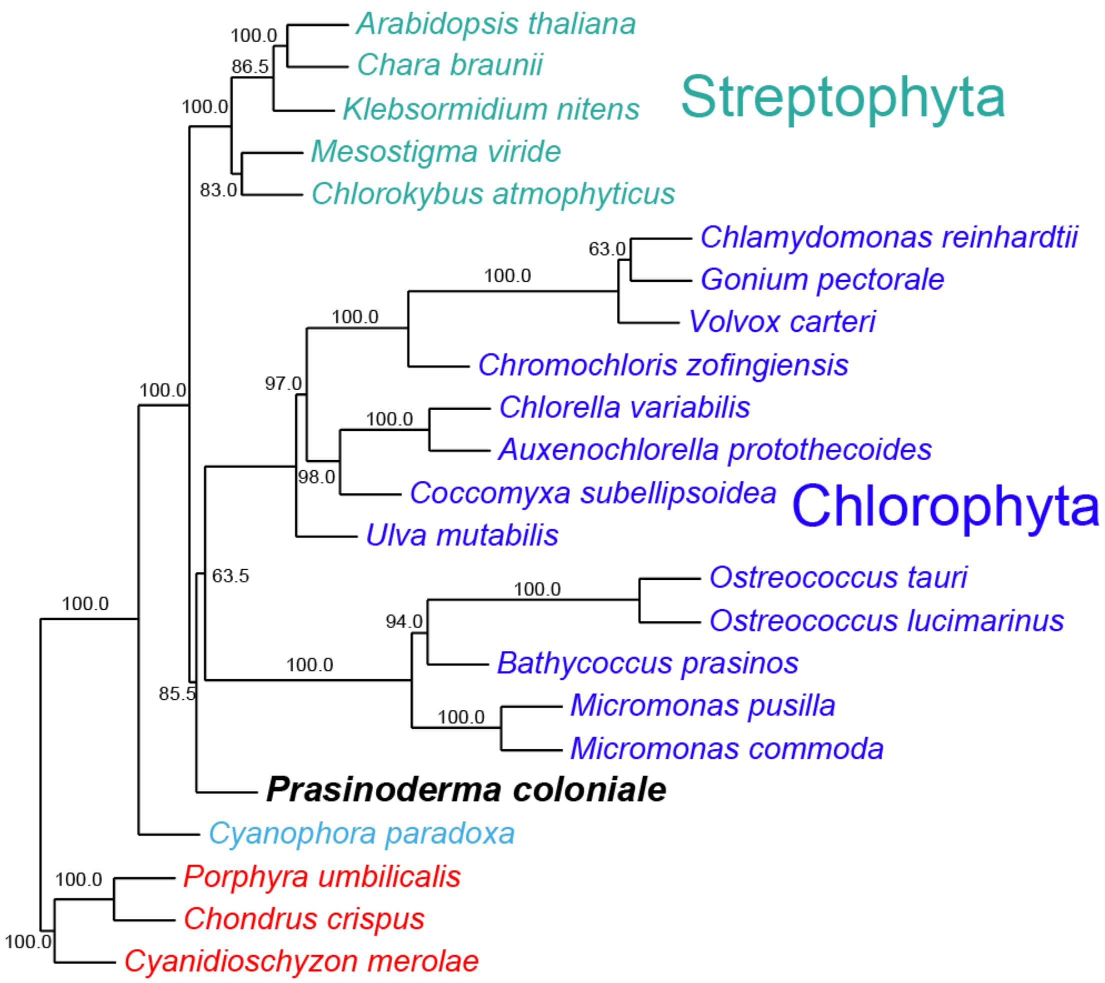

Supplementary Figure 7. The coalescent phylogenetic tree of 23 species with 45 single-copy genes using ASTRAL place Prasinodermophyta (*Prasinoderma coloniale*) to the sister lineage of other Chlorophyta with high support (85.5).

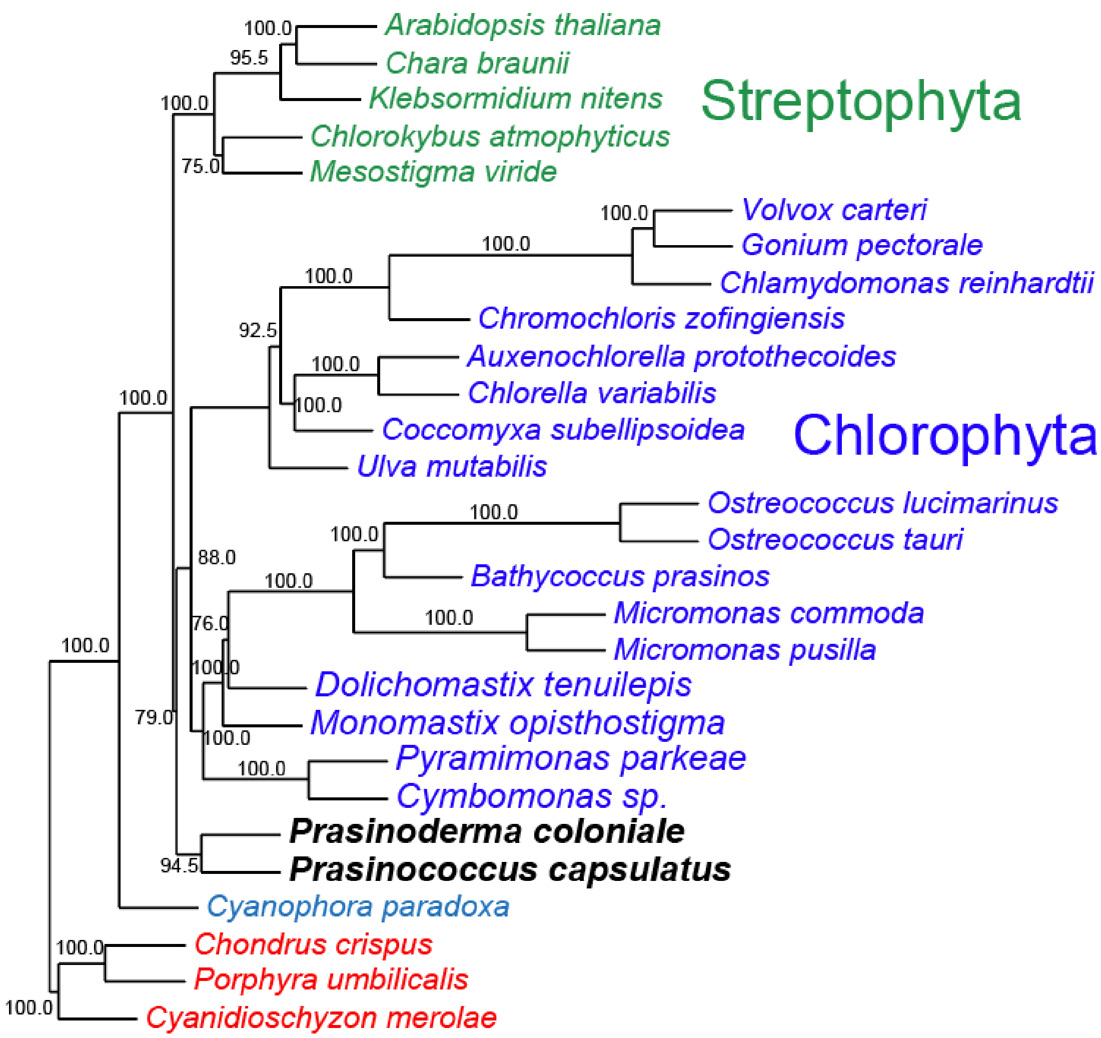

Supplementary Figure 8. The coalescent phylogenetic tree of 28 species with 213 single-copy genes using ASTRAL place *Prasinoderma coloniale* to the sister lineage of other Chlorophyta with relatively lower support (79.0). In order to increase the number of single-copy genes to construct the phylogenetic tree, we allowed gene absence up to 8 species in one gene family. As for the species, 5 transcriptome data were added including *Prasinococcus capsulatus*, *Cymbomonas sp.*, *Pyramimonas parkeae*, *Monomastix opisthostigma*, *Dolichomastix tenuilepis* but removed the *Ostreococccus sp*. RCC809.

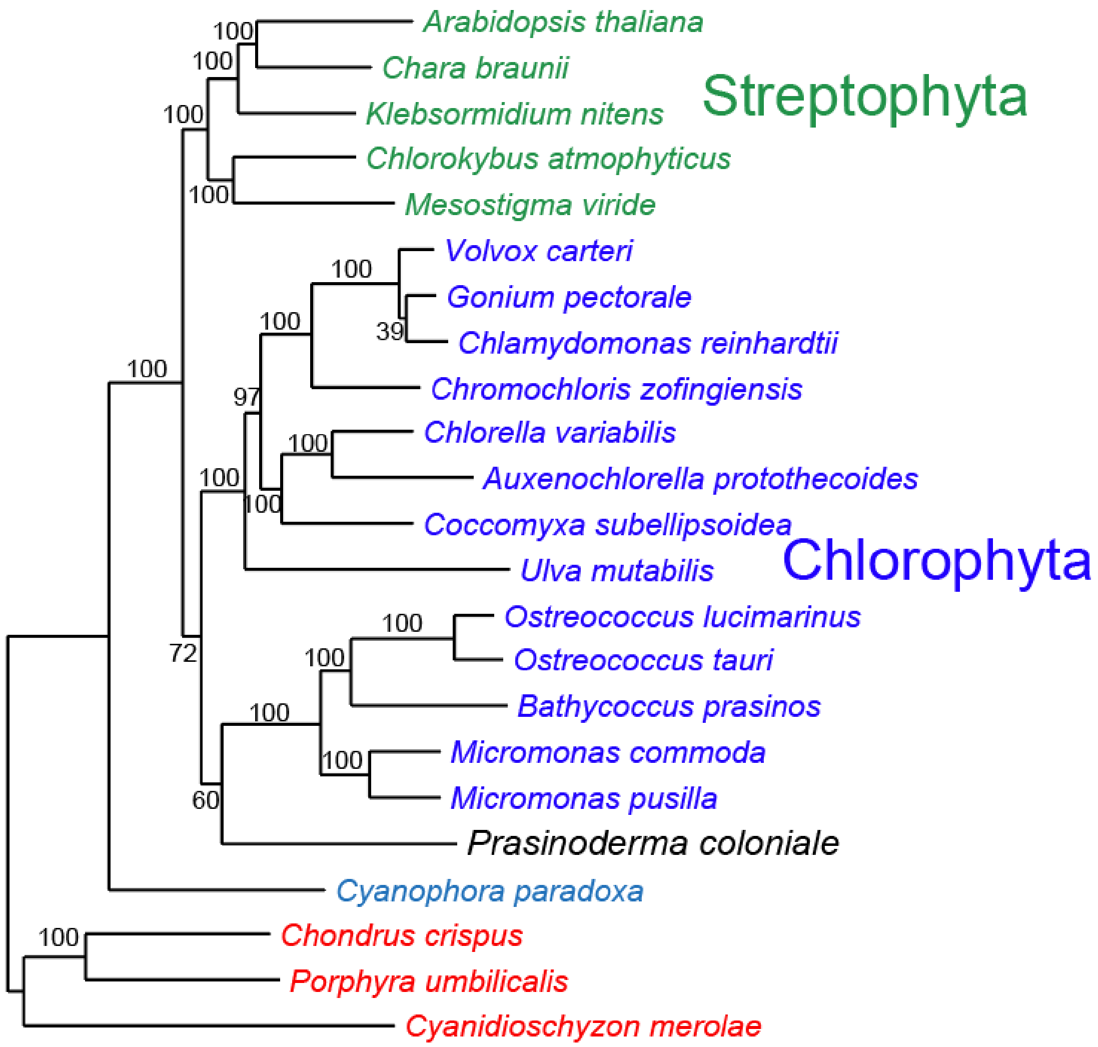

Supplementary Figure 9. The concatenated phylogenetic tree of 23 species with 45 single-copy genes using RAxML place *Prasinoderma coloniale* to the sister lineage of Mamiellales (Mamiellophyceae). PROTCATGTR AA substitution model and 500 bootstraps were used in phylogenetic analysis. The Prasinodermophyta, represented only by *Prasinoderma coloniale*, was resolved as sister to the Mamiellales (Mamiellophyceae) with high support (81). This artificial placement (i.e. *Prasinoderma coloniale* diverging within the Chlorophyta) gained high support by bootstrapping (100).

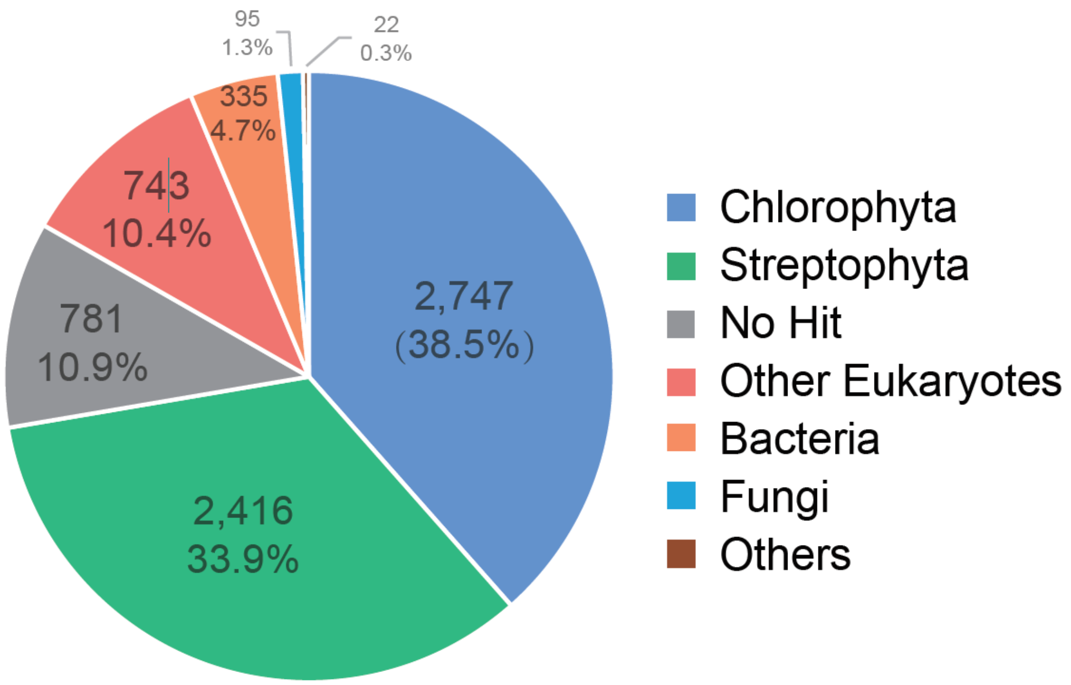

Supplementary Figure 10. Taxon distribution of the best BLAST hits of the *P. coloniale* genes in the NCBI non-redundant protein database.

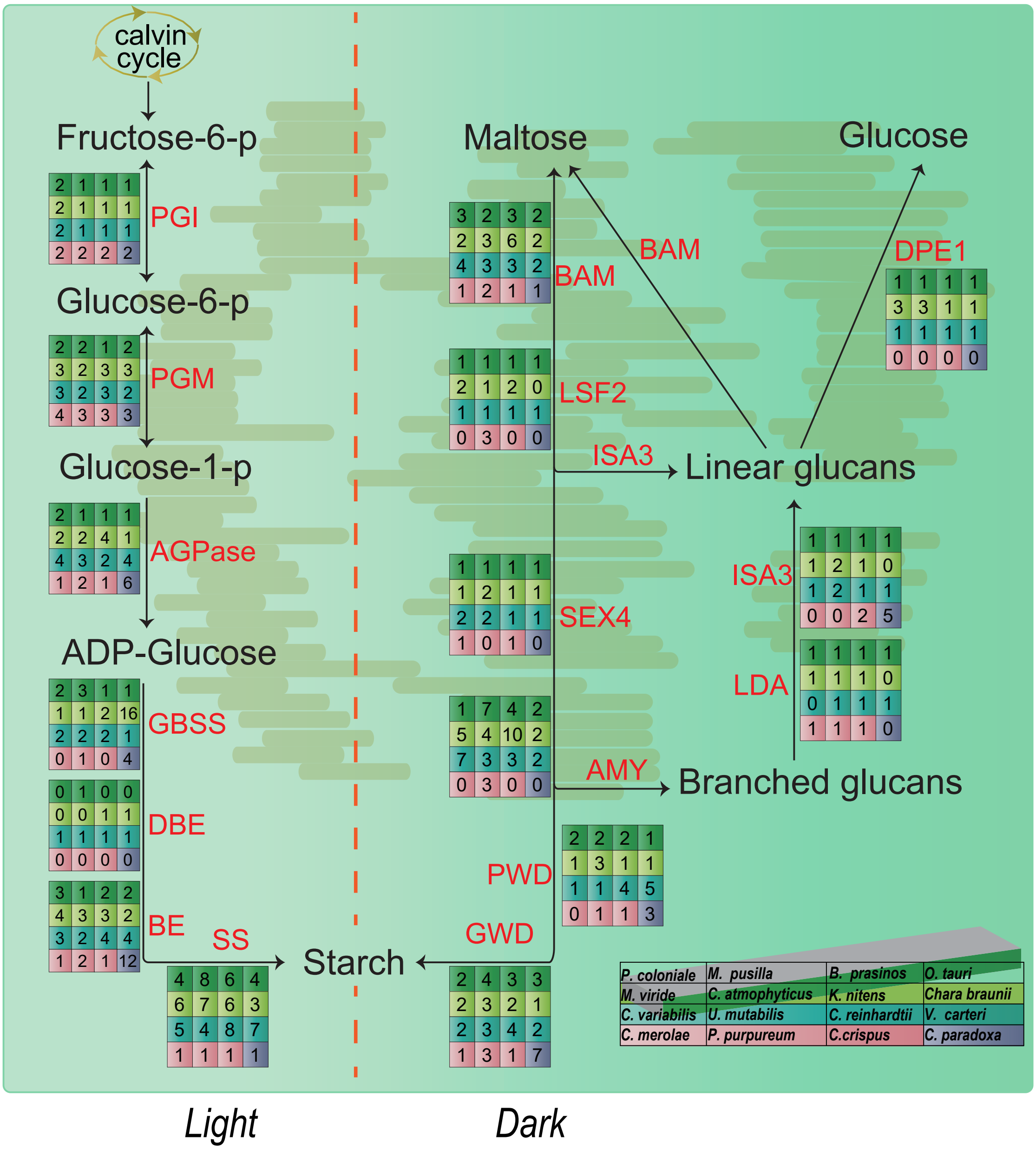

Supplementary Figure 11. Reconstruction the Pathways of starch synthesis and degradation in 16algae genomes. Fructose-6-p, fructose 6-phosphate; Glucose-6-p, glucose 6-phosphate; Glucose-1-p, glucose 1-phosphate; ADP-Glucose, ; PGI, phosphoglucose iosomerase; PGM, hosphoglucomutase; AGPase, ADP-glucose pyrophosphorylase; GBSS, granule-bound starch synthase; DBE, debranching enzyme; BE, branching enzyme; SS, Starch synthase; GWD, α-glucan,water dikinase; PWD, phosphoglucan,water dikinase; AMY, α-amylase; SEX4, Phosphoglucan phosphatase DSP4; ISA3, Isoamylase 3; LSF2, Phosphoglucan phosphatase LSF2; BAM, β-amylase; DPE1, Glucanotransferase (dpe1); LDA, Protein LIMIT DEXTRINASE; the gene copy number of each species is shown in the Supplementary Table 16.

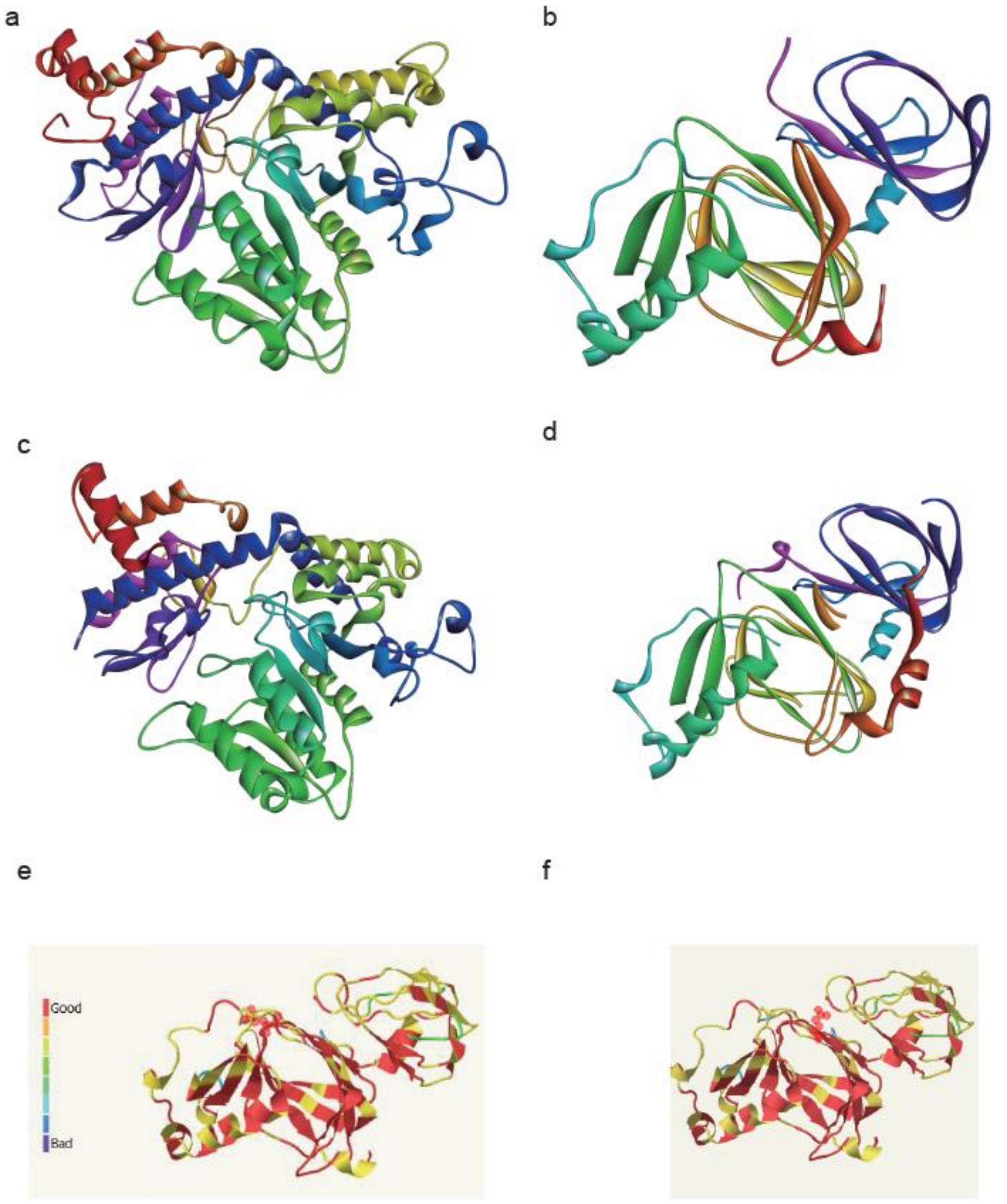

Supplementary Figure 12. Structural Features of KYU and HAAO Protein Domains Constructed by Swiss-Model Homology-Based Approach. a) KYU domain based on the crystal structure of *P. coloniale*. b) HAAO domain based on the crystal structure of *P. coloniale*. c) KYU domain based on the crystal structure of human. d). HAAO domain based on the crystal structure of human. e) KYU domain alignment of a and c. f) HAAO domain alignment of b and d.

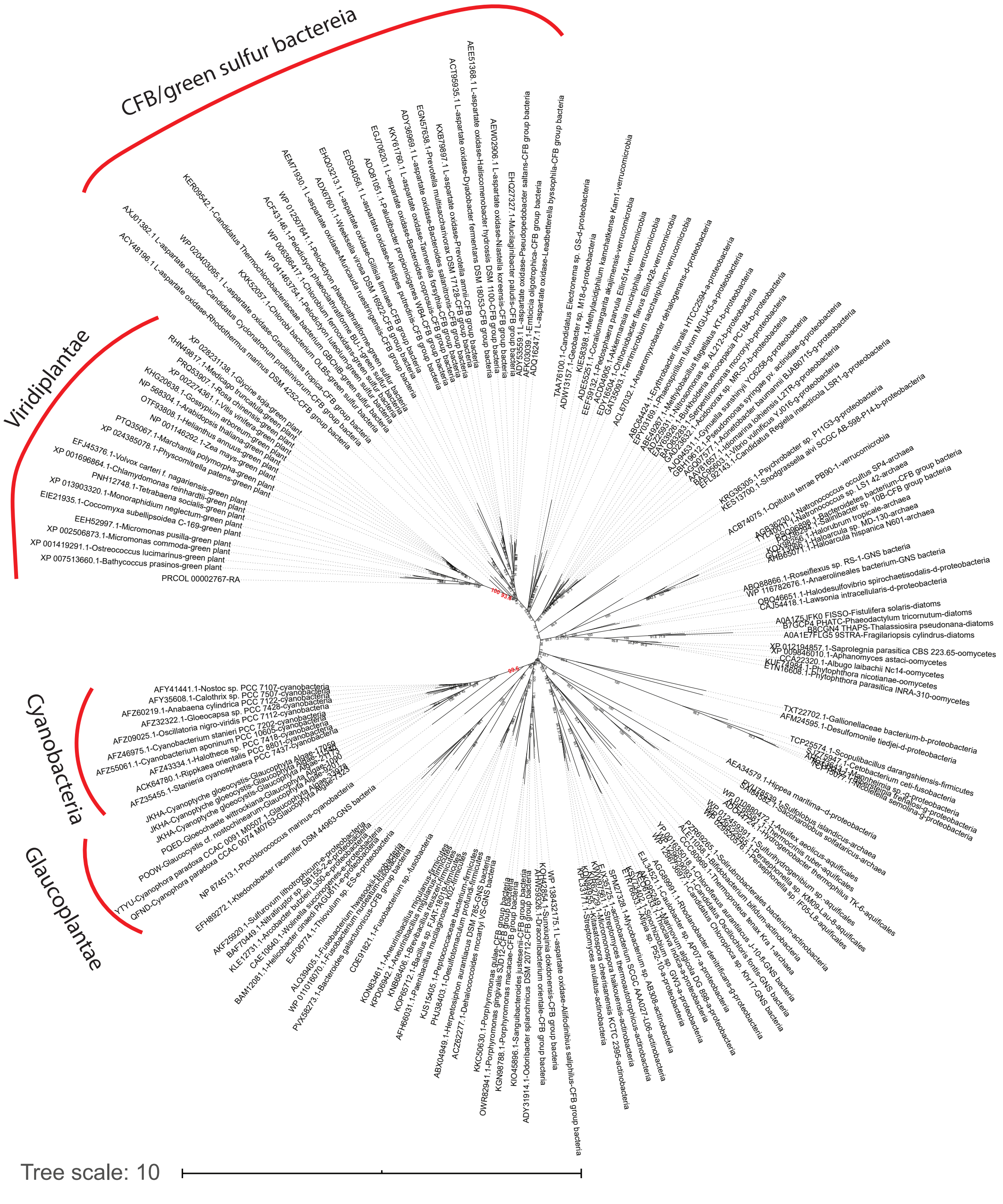

Supplementary Figure 13. The phylogenetic tree of AO (Aspartate oxidase) based on IQ-TREE method, using the best model predicted by IQ-TREE with 5000 replicates. The important bootstraps are highlighted by the red color.

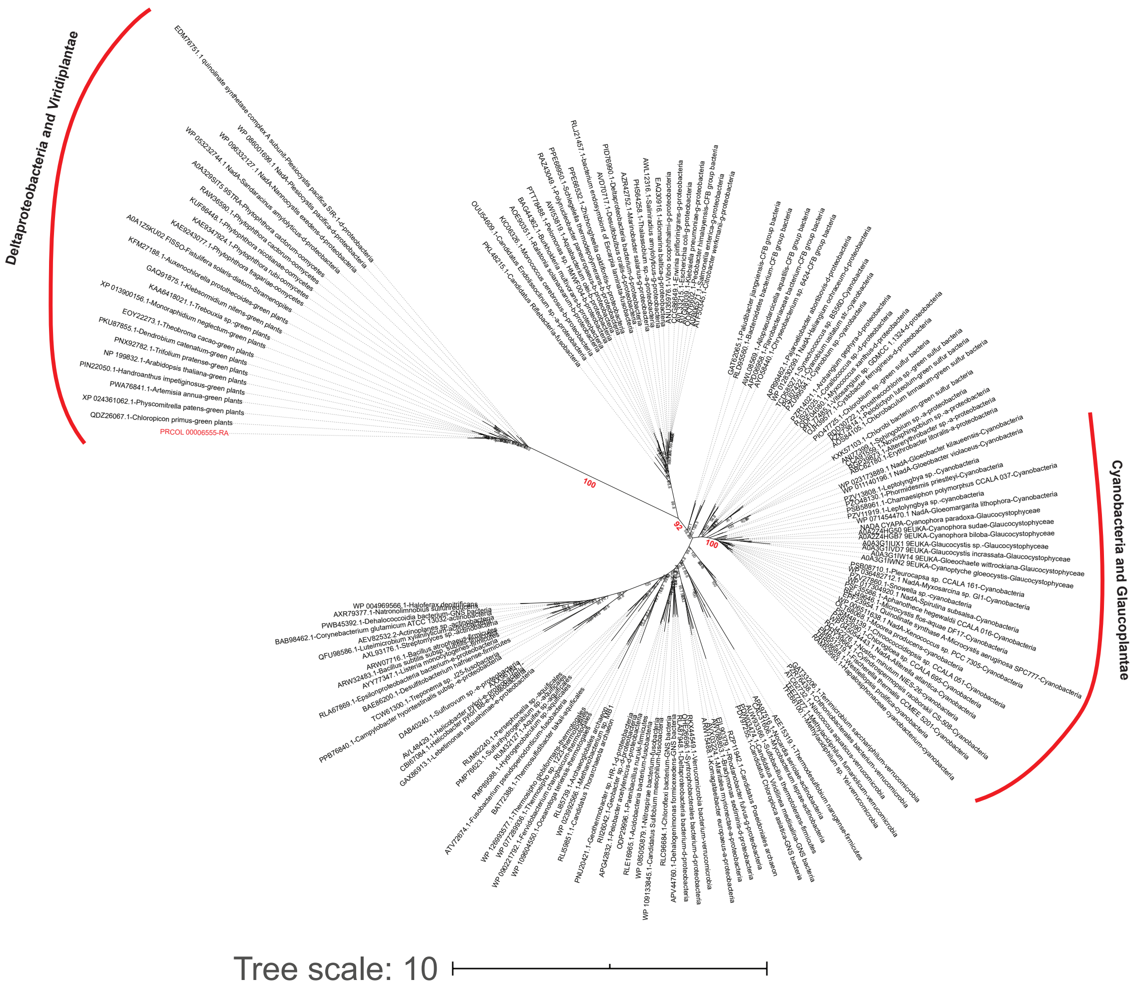

Supplementary Figure 14. The phylogenetic tree of QS (Quinolinate synthase) based on IQ-TREE method, using the best model predicted by IQ-TREE with 5000 replicates. The important bootstraps are highlighted by the red color.

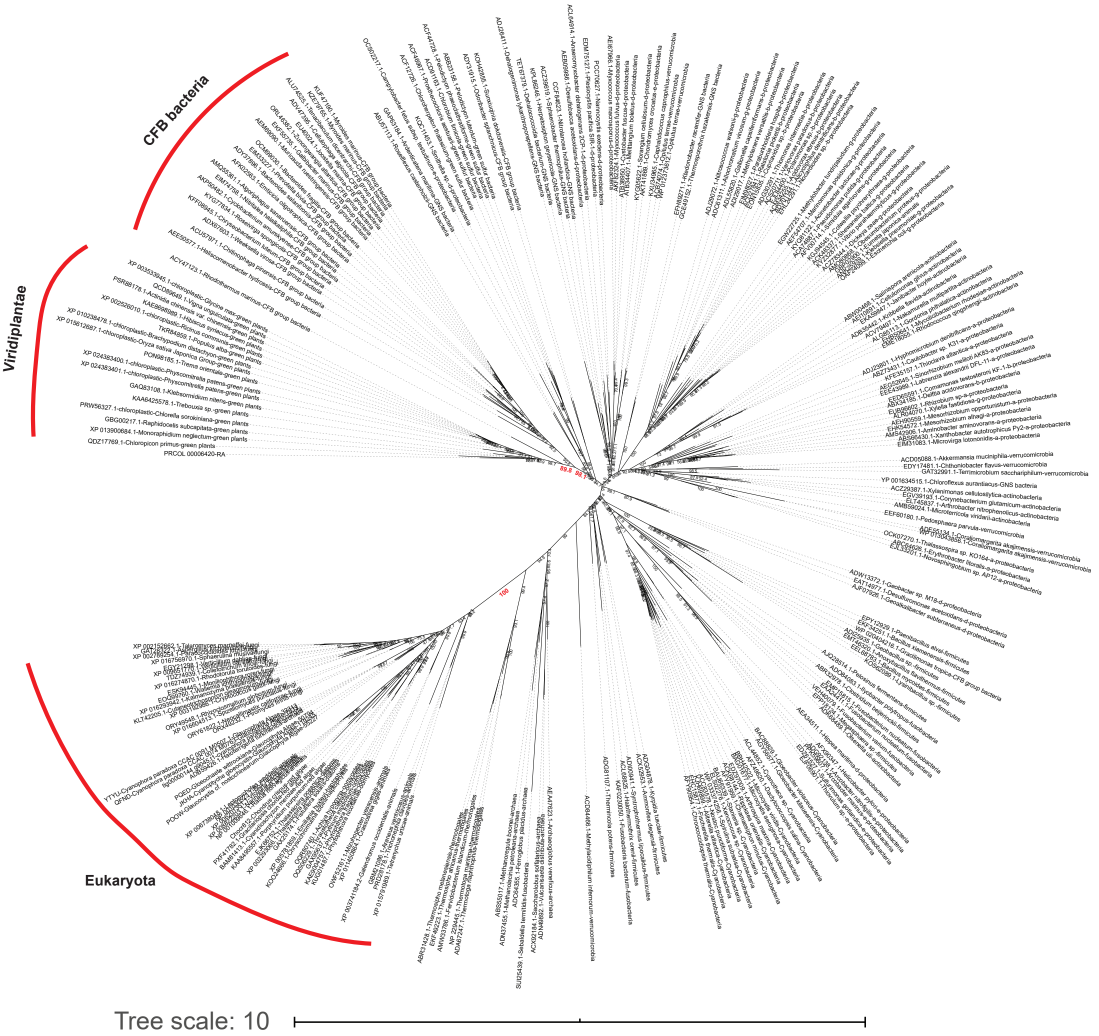

Supplementary Figure 15. The phylogenetic tree of NNP (nicotinate nucleoside pyrophosphatase) based on IQ-TREE method, using the best model predicted by IQ-TREE with 5000 replicates. The important bootstraps are highlighted by the red color.

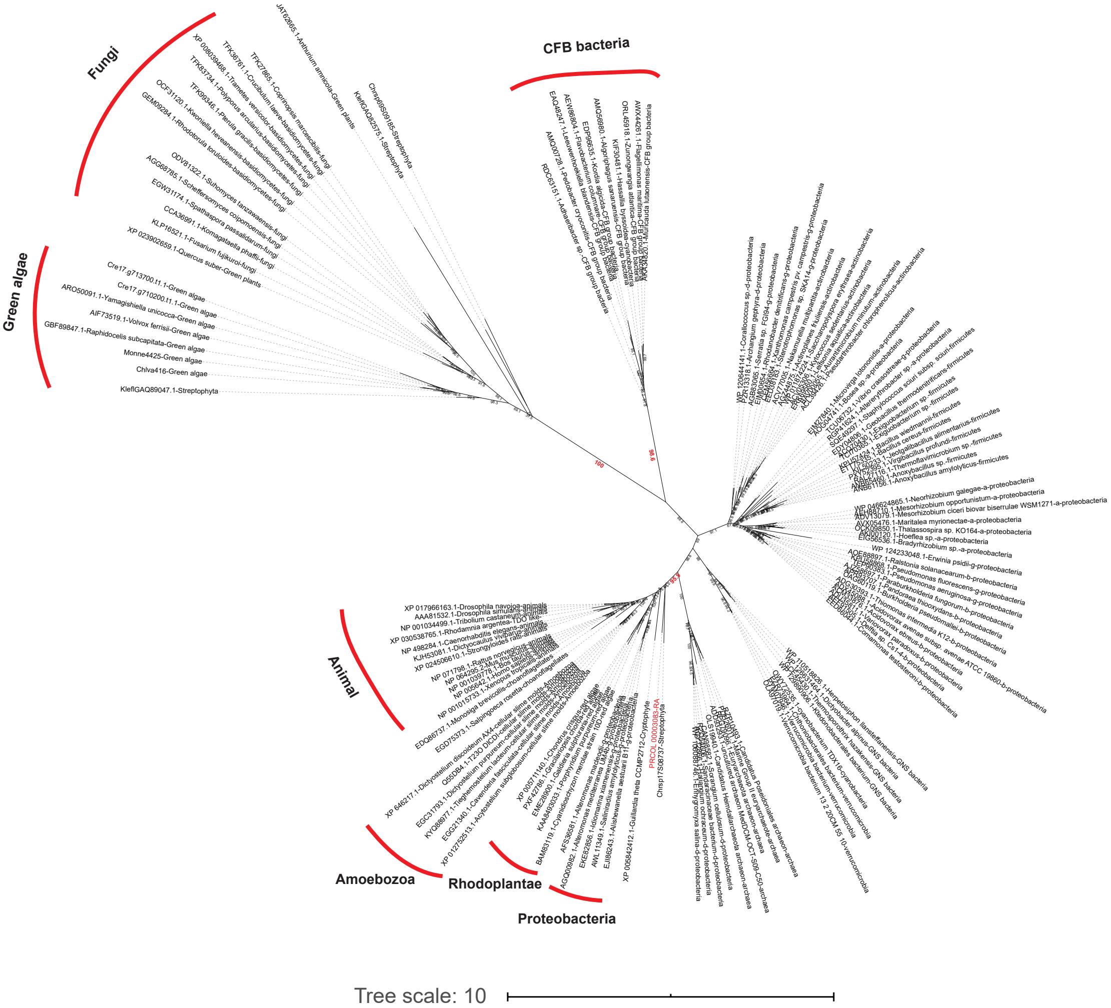

Supplementary Figure 16. The phylogenetic tree of TDO/IDO (Tryptophan-/ Indoleamine 2,3-dioxygenase) based on IQ-TREE method, using the best model predicted by IQ-TREE with 5000 replicates. The important bootstraps are highlighted by the red color.

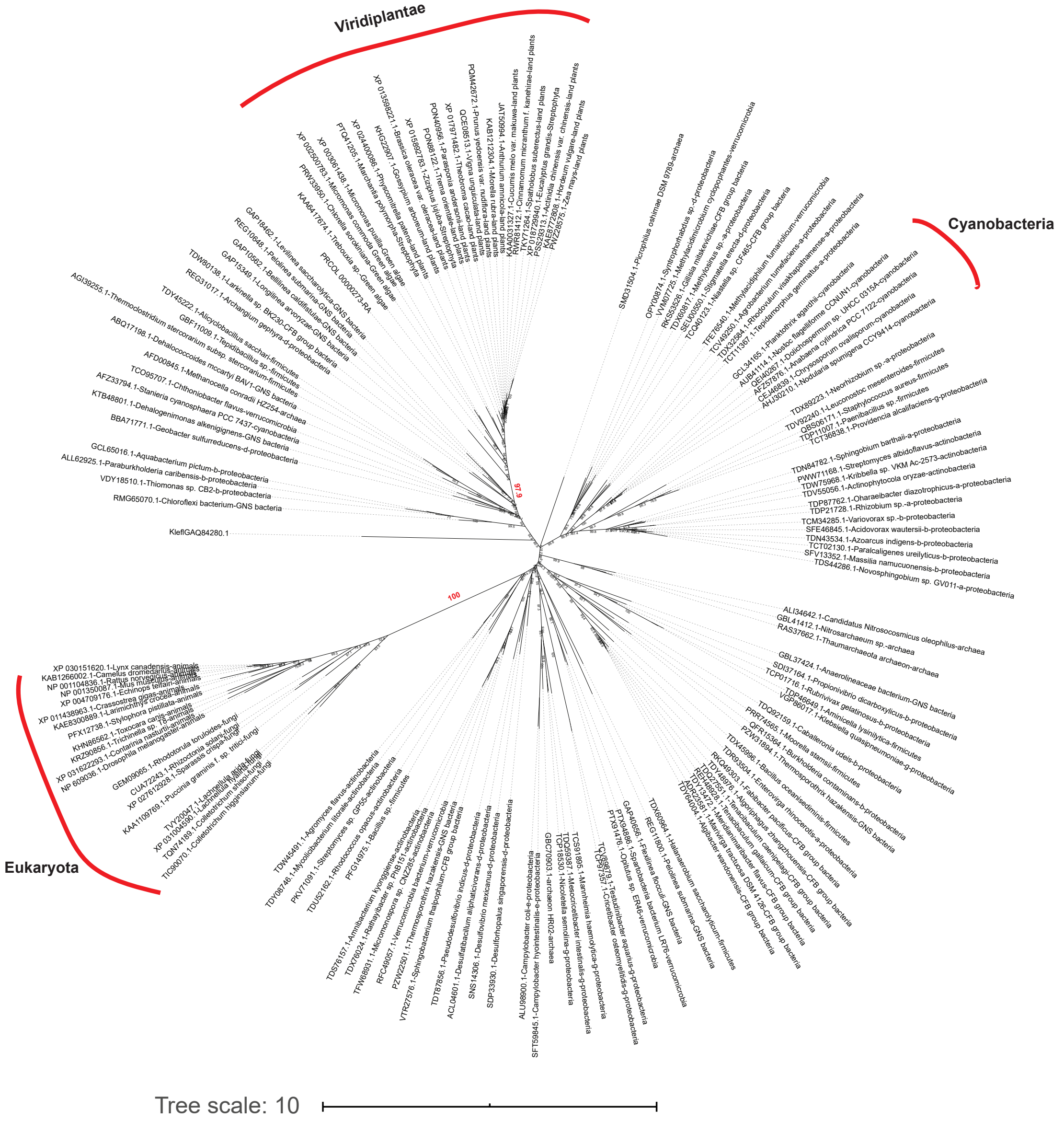

Supplementary Figure 17. The phylogenetic tree of AFM (Arylformamidase) based on IQ-TREE method, using the best model predicted by IQ-TREE with 5000 replicates. The important bootstraps are highlighted by the red color.

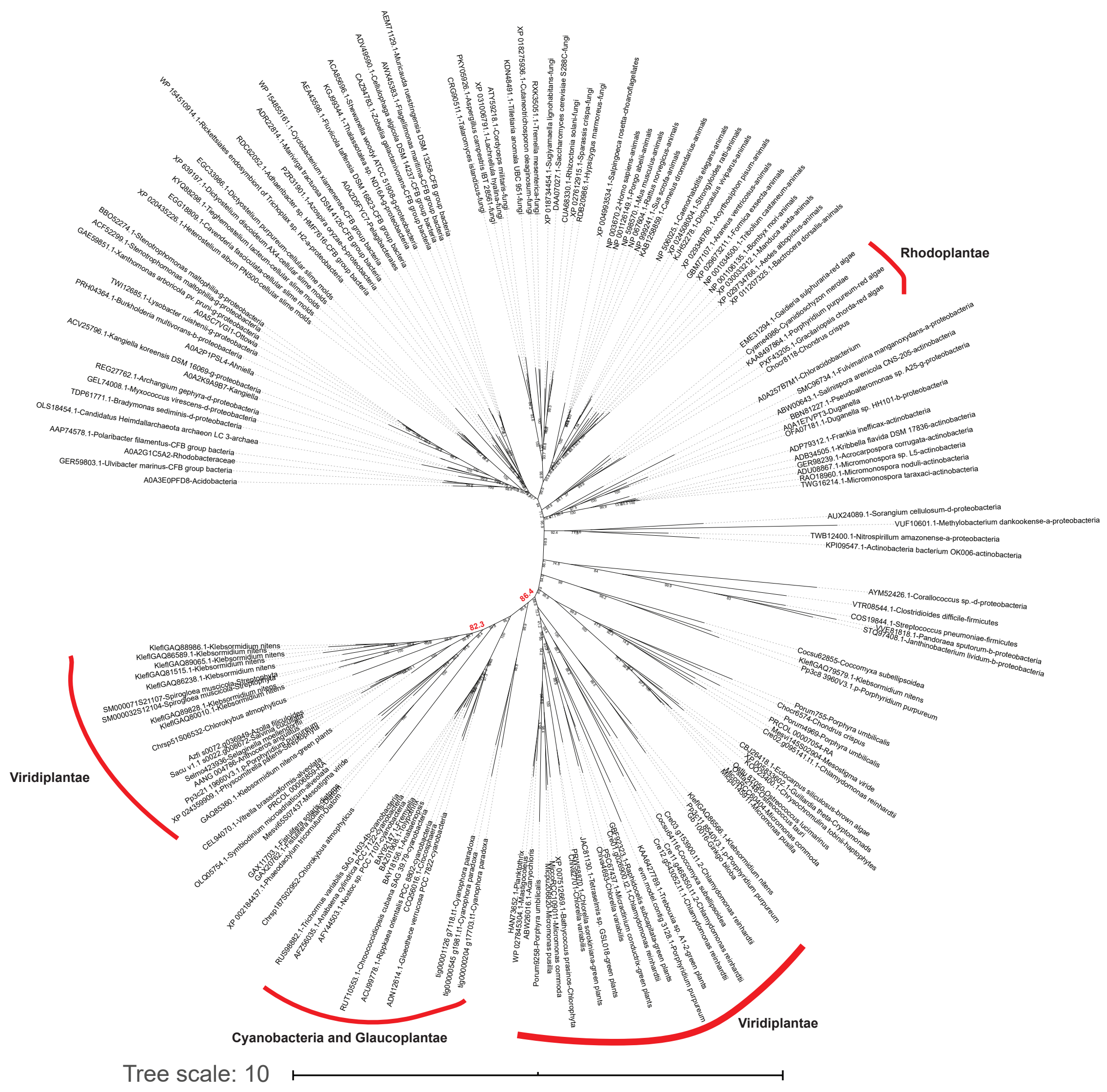

Supplementary Figure 18. The phylogenetic tree of KMO (Kynurenine 3-monooxygenase) based on IQ-TREE method, using the best model predicted by IQ-TREE with 5000 replicates. The important bootstraps are highlighted by the red color.

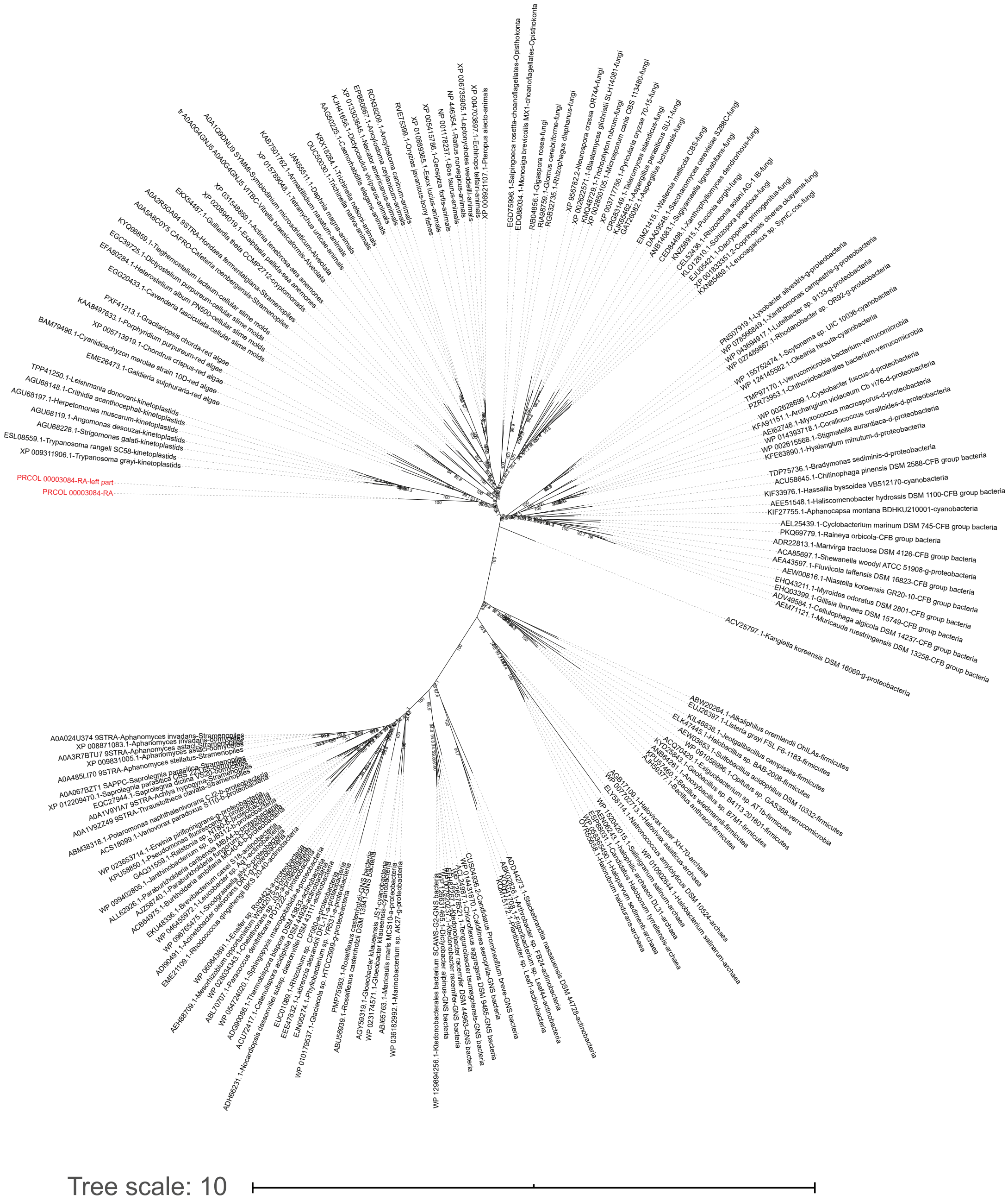

Supplementary Figure 19. The phylogenetic tree of KYU (kynureninase) based on IQ-TREE method, using the best model predicted by IQ-TREE with 5000 replicates. The important bootstraps are highlighted by the red color.

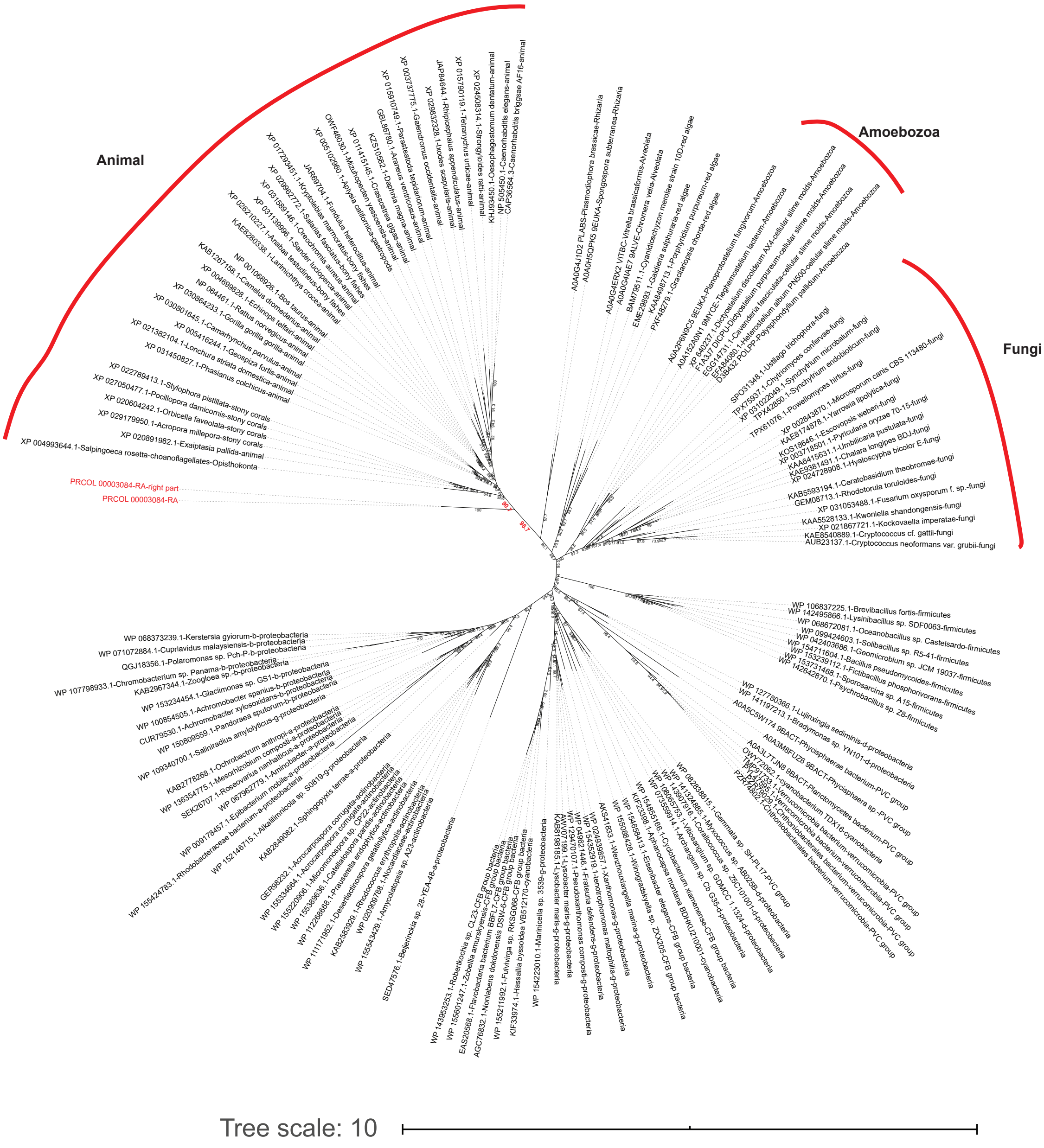

Supplementary Figure 20. The phylogenetic tree of HAAO (3-hydroxyanthranilate 3,4-dioxygenase) based on IQ-TREE method, using the best model predicted by IQ-TREE with 5000 replicates. The important bootstraps are highlighted by the red color.

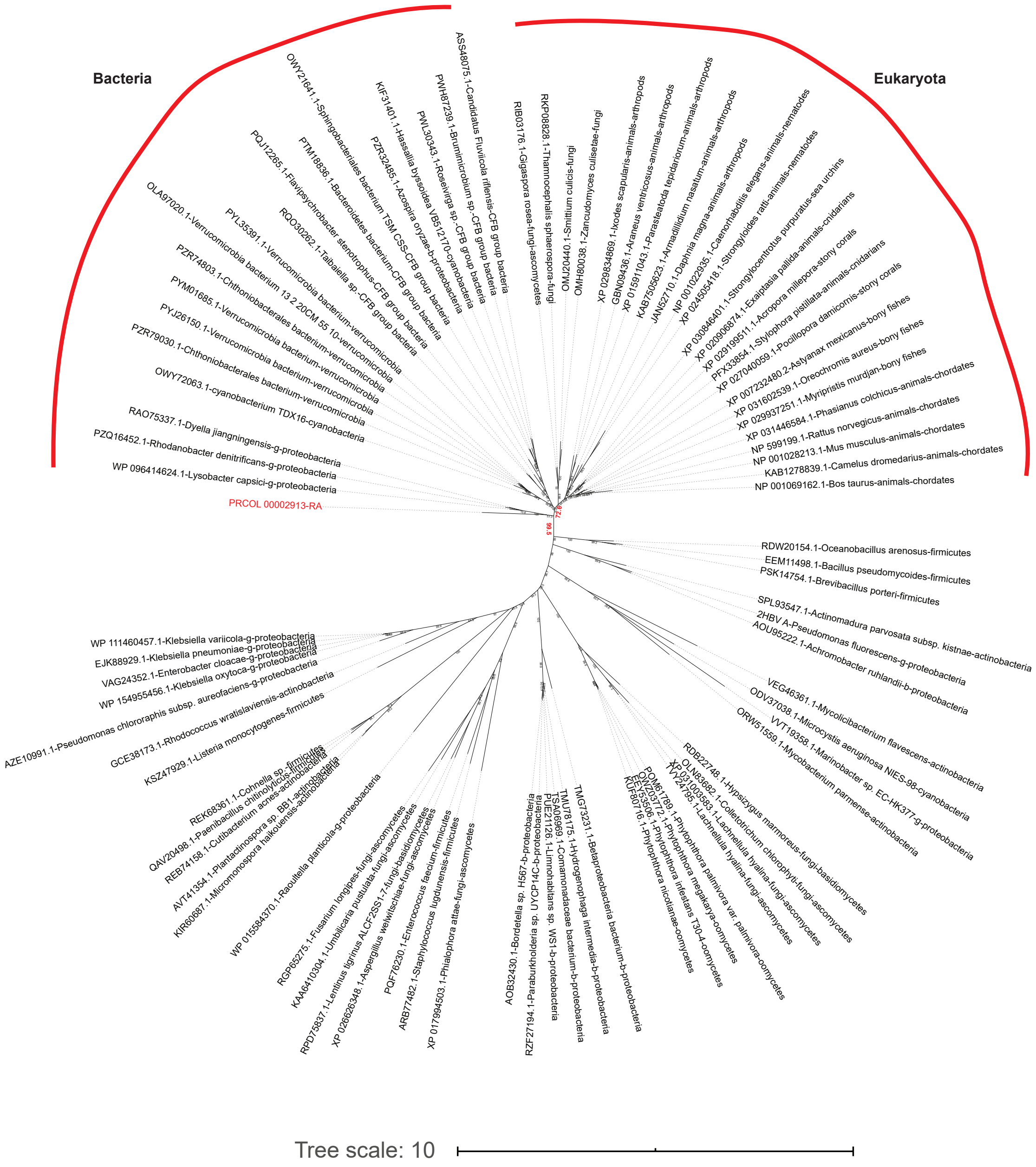

Supplementary Figure 21. The phylogenetic tree of ACMSD (amino-β-carboxymuconate-semialdehyde-decarboxylase) based on IQ-TREE method, using the best model predicted by IQ-TREE with 5000 replicates. The important bootstraps are highlighted by the red color.
