# Supplementary material for: The genome of Prasinoderma coloniale unveils the existence of a third phylum within green plants
Source: Nat Ecol Evol. 2020 Jun 22;4(9):1220–31. doi: 10.1038/s41559-020-1221-7 (PMC7455551; doi:10.1038/s41559-020-1221-7)
Supplement: Supplementary file 2 — Reporting Summary [file 41559_2020_1221_MOESM2_ESM.pdf]

nature research

Corresponding author(s): Huan Liu

Last updated by author(s): Apr 30, 2020

Reporting Summary

Nature Research wishes to improve the reproducibility of the work that we publish. This form provides structure for consistency and transparency in reporting. For further information on Nature Research policies, see Authors & Referees and the Editorial Policy Checklist.

Statistics

For all statistical analyses, confirm that the following items are present in the figure legend, table legend, main text, or Methods section.

n/a	Confirmed	
☒	☐	The exact sample size (*n*) for each experimental group/condition, given as a discrete number and unit of measurement
☒	☐	A statement on whether measurements were taken from distinct samples or whether the same sample was measured repeatedly
☒	☐	The statistical test(s) used AND whether they are one- or two-sided<br>*Only common tests should be described solely by name; describe more complex techniques in the Methods section.*
☒	☐	A description of all covariates tested
☒	☐	A description of any assumptions or corrections, such as tests of normality and adjustment for multiple comparisons
☒	☐	A full description of the statistical parameters including central tendency (e.g. means) or other basic estimates (e.g. regression coefficient) AND variation (e.g. standard deviation) or associated estimates of uncertainty (e.g. confidence intervals)
☐	☒	For null hypothesis testing, the test statistic (e.g. *F*, *t*, *r*) with confidence intervals, effect sizes, degrees of freedom and *P* value noted<br>*Give P values as exact values whenever suitable.*
☒	☐	For Bayesian analysis, information on the choice of priors and Markov chain Monte Carlo settings
☒	☐	For hierarchical and complex designs, identification of the appropriate level for tests and full reporting of outcomes
☒	☐	Estimates of effect sizes (e.g. Cohen's *d*, Pearson's *r*), indicating how they were calculated

Our web collection on statistics for biologists contains articles on many of the points above.

Software and code

Policy information about availability of computer code

Data collection

Paired-end libraries with insert sizes of 170 bp, 250 bp, 2 kb, 5 kb, 10 kb and 20 kb were constructed following standard Illumina protocols. The libraries were sequenced on an Illumina HiSeq 2000/4000. A total of 179Gb (about 8885.94X) paired-end data were generated for Prasinoderma coloniale (CCMP 1413). Besides, 7.4 Gb Pacbio long reads were generated by Sequel II platform.

For Illumina sequencing, we considered two ways of library construction. The rRNA-depleted RNA library was constructed using the ribo-zero rRNA removal kit (plant) (Illumina, American) following the manufacturer's protocol, while the poly (A)-selected RNA library was constructed using the ScriptSeq Library Prep kit (Plant leaf) (Illumina, American) following the manufacturer's protocol.

Data analysis

The list of Software used in this study are as follows:

CLC Assembly Cell (version 5.0.1)
Pairfq (version 0.16.0)
SOAPfilter (version 2.2)
fastp (version v0.20.1)
Kmerfreq (version 1.0)
Jellyfish (version v2.3.0)
SPAdes (version 3.10.1)
SSPACE (version 3.0)
GapCloser (version 1.12)
MeDuSa (version 1.6)
NextDenovo (version v2.2)
NextPolish (version v1.1.0)
BUSCO (version3)
Soap (version 2.21)
blat (v36)

Bridger_r2014-12-01
Trinityrnaseq (version 2.1.1)
Tophat2 (version 2.1.0)
RepeatModeler (version 1.0.8)
GenomeTools (version 1.5.8)
MITE-hunter
LTRharvest
PASApipeline-2.1.0
AUGUSTUS (version 3.2.3)
GeneMark (version 1.0)
MAKER (version 2.31.8)
SNAP (version 2006-07-28)
Samtools (version 0.1.19)
blast-2.2.26
ncbi-blast-2.2.31+
Blast2go (version 2.5.0)
InterProScan 5.28-67.0
OrthoFinder (version 1.1.8)
MAFFT (version 7.310)
RAxML (version 8.2.4)
IQ-tree (version 1.6.1)
ASTRAL (version 4.11.1)

For manuscripts utilizing custom algorithms or software that are central to the research but not yet described in published literature, software must be made available to editors/reviewers. We strongly encourage code deposition in a community repository (e.g. GitHub). See the Nature Research guidelines for submitting code & software for further information.

Data

Policy information about availability of data

All manuscripts must include a data availability statement. This statement should provide the following information, where applicable:

- Accession codes, unique identifiers, or web links for publicly available datasets
- A list of figures that have associated raw data
- A description of any restrictions on data availability

The whole genome assemblies for P. coloniale in this study are deposited at DDBJ/ENA/GenBank under the accession numbers of RQSC00000000. Those data are also available in the CNGB Nucleotide Sequence Archive (CNSA: http://db.cngb.org/cnsa; accession number CNA0002354).

Field-specific reporting

Please select the one below that is the best fit for your research. If you are not sure, read the appropriate sections before making your selection.

☒ Life sciences ☐ Behavioural & social sciences ☐ Ecological, evolutionary & environmental sciences

For a reference copy of the document with all sections, see nature.com/documents/nr-reporting-summary-flat.pdf

Life sciences study design

All studies must disclose on these points even when the disclosure is negative.

Sample size	Axenic cultures of Prasinoderma coloniale (CCMP 1413) were obtained from the Culture Collection of Algae at the University of Cologne and grown in a modified ASP12 culture medium (http://www.ccac.uni-koeln.de/).
Data exclusions	The reads with low quality are more likely to contain errors, which might complicate the assembly process, and were excluded. Detailed criteria are provided in the subsection of Method "Genome sequencing and assembly"
Replication	NA
Randomization	No randomization is required for our experiment.
Blinding	Blind experiment is not required for our work.

Reporting for specific materials, systems and methods

We require information from authors about some types of materials, experimental systems and methods used in many studies. Here, indicate whether each material, system or method listed is relevant to your study. If you are not sure if a list item applies to your research, read the appropriate section before selecting a response.

Materials & experimental systems

n/a	Involved in the study
☒	☐ Antibodies
☒	☐ Eukaryotic cell lines
☒	☐ Palaeontology
☒	☐ Animals and other organisms
☒	☐ Human research participants
☒	☐ Clinical data

Methods

n/a	Involved in the study
☒	☐ ChIP-seq
☒	☐ Flow cytometry
☒	☐ MRI-based neuroimaging
