# Supplementary material for: The genome of Prasinoderma coloniale unveils the existence of a third phylum within green plants
Source: Nat Ecol Evol. 2020 Jun 22;4(9):1220–31. doi: 10.1038/s41559-020-1221-7 (PMC7455551; doi:10.1038/s41559-020-1221-7)
Supplement: Supplementary file 4 — Taxonomic Acts and Revisions. [file 41559_2020_1221_MOESM4_ESM.zip › Taxonomic Acts and Revisions 1/NHS_Chloro+Strepto_D4_G4.pdf]

rRNA Synapomorphies of the **Chlorophyta** + **Streptophyta** clade, to the exclusion of **Prasinodermophyta**, Glaucoplantae and Rhodoplantae

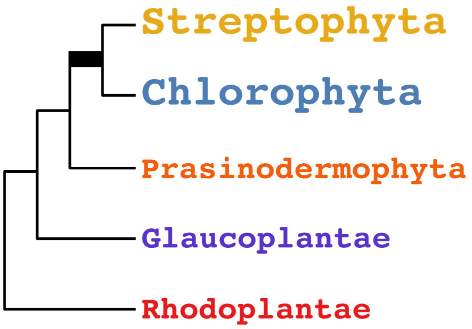

28S rRNA - Helix G4 [H2093]

reverse strand - unpaired nucleotide between bp 10 and 11

NHS (non homoplasious synapomorphy)

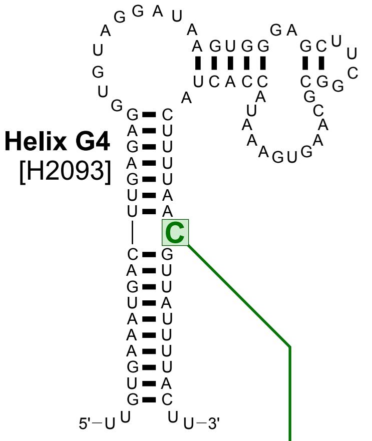

28S rRNA - Helix D4 [H628]

bp 2: U-G ==> **G-C**

homoplasies in the Chlorophyta [Viridiplantae]:
in the Pedinomonadales and Pseudoscourfieldiales,
the base pair reverted to the plesiomorphic state (U-G)

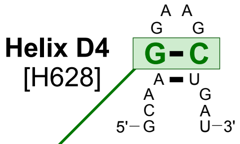


```
Arabidopsis_thaliana              GGC-GCA-A-G-GAAG-C-U-G-//-GUGAAAUGACUUGAGAG-GUGUAGGAUA-AGUGG-GA-GC----UUCG----GC-GCAAGUGAAAUA-CCACU-A-CUUUU-AA-C-GUUAUUUUAC-UUA
Pinus_taeda                       GGC-GCA-A-G-GAAG-C-U-G-//-GUGAAAUGACUUGAGAG-GUGUAGAAUA-AGUGG-GA-GCCG--AUUU--UGGC-ACAAGUGAAAUA-CCACU-A-CUUUU-AA-C-GUUAUUUUAC-UUA
Huperzia_selago                   GGC-GCA-A-G-GAAA-C-U-G-//-GUGAAAUGACUUGAGAG-GUGUAGAAUA-AGUGG-GA-GC----UCCG----GC-GCAAGUGAAAUA-CCACU-A-CUUUU-AA-C-GUUAUUUUAC-UUA
Polytrichum_juniperinum           GGC-GCA-A-G-GAAC-C-U-G-//-GUGAAAUGACUUAAGAG-GUGUAGAAUA-AGUGG-GA-GC-----/------GC-GAAAGUGAAAUA-CCACU-A-CUUUU-AA-C-GUUAUUUUAC-UUA
Marchantia_polymorpha             GGC-GCA-A-A-UAAC-U-U-A-//-GUGGAAUGACUCAAGAG-GUGUAGAAUA-AGUGG-GA-GGUCC-GUGA-GGGCC-GCAAGUGAAAUA-CCACU-A-CUUUU-GA-C-GUUAUUUUAC-UUA
Nothoceros_vincentianus           GGC-GUA-A-G-GAAC-C-U-G-//-GUGAAAUGACUUAAGAG-GUGUAGAAUA-AGUGG-GA-GCC---GUA----GGC-GCAAGUGAAAUA-CCACU-A-CUUUU-AA-C-GUUAUUUUAC-UUA
Mesotaenium_endlicherianum        GGC-GCA-A-G-UAAC-C-U-G-//-GUGAAAUGACUUAAGAG-GUGUAGAAUA-AGUGG-GA-GGC---UUU----GCC-GAAAGUGAAAUA-CCACU-A-CUUUU-AA-C-GUUAUUUUAC-UUA
Cosmarium_ochthodes               GGC-GUA-G-G-UAAC-C-C-G-//-GUGAAAUGACUCAGGAG-GUGUAGAAUA-AGUGG-GA-GG----GUGA----CC-GCAAGUGAAAUA-CCACU-A-CUCUU-GA-C-GUUAUUUUAC-UUA
Coleochaete_scutata               GGC-GCA-A-G-UAAC-C-U-G-//-GUGAAAUGACUUGAGAG-GUGUAGAAUA-AGUGG-GA-GC----UCCG----GC-GCAAGUGAAAUA-CCACU-A-CUUUC-AA-C-GUUAUUUUAC-UUA
Chaetosphaeridium_globosum        GGC-GCA-A-G-GAAG-C-U-G-//-GUGAAAUGACUUAAGAG-GUGUAGAAUA-AGUGG-GA-GC----UCCG----GC-GCAAGUGAAAUA-CCACU-A-CUUUU-AA-C-GUUAUUUUAC-UUA
Chara_braunii                     GGC-GGA-A-G-AAAC-C-U-G-//-GUGAAAUGACUUGAGAG-GUGUAGAAUA-AGUGG-GA-GCC---UUAC---GGC-GCAAGUGAAAUA-CCACU-A-CUUUU-AA-C-GUUAUUUUAC-UUA
Klebsormidium_nitens              GGC-GCA-A-G-UAAC-C-U-G-//-GUGAAAUGACUUAAGAG-GUGUAGAAUA-AGUGG-GA-GC----UUCG----GC-GCAAGUGAAAUA-CCACU-A-CUUUU-AA-C-GUUAUUUUAC-UUA
Chlorokybus_atmophyticus          GGC-GCA-A-G-GAAA-C-U-G-//-GUGAACUGGAUUGGGAG-GUGUAGAAUA-AGUGG-GA-GC----UUCG----GC-GCCGUUGAAAUA-CCACU-A-CUCCC-AA-C-UCCGGUUUGC-UUA
Spirotaenia_condensata            GGC-GCA-A-G-GAAA-C-U-G-//-GUGAACUGGAAUGGGGG-GUGUAGAAUA-AGUGG-GA-GC----UUCG----GC-GCCAGUGAAAUA-CCACU-A-CCCCU-AU-C-UCCGGUUUGC-UUA
Mesostigma_viride                 GGC-GCA-A-G-GAAA-G-U-G-//-GUGAACUGGAUCGGGAG-GUGUAGCAUA-AGUGG-GA-GC----UCGC----GC-GACCUUGAAAUA-CCACU-A-CUCCC-GA-C-UCCGGUUUGC-UUA

Ulva_mutabilis                    AGC-GCA-A-G-GAAA-C-U-G-//-GUGAAAUGACUUGAGAG-GUGUAGCAUA-AGUGG-GA-GC----UUCG----GC-GCCAGUGAAAUA-CCACU-A-CUUUU-AA-C-GUUAUUUUAC-UUA
Chlamydomonas_reinhardtii         AGC-GCA-A-G-UAAC-C-U-G-//-GUGAAAUAACUUAAGAG-GUGUAGAAUA-AGUGG-GA-GC----UUCG----GC-GACGGUGAAAUA-CCACU-A-CUUUU-AA-C-GUUGUUUUAC-UUA
Volvox_carteri                    AGC-GCA-A-G-UAAC-C-U-G-//-GUGAAAUAACUUAAGAG-GUGUAGAAUA-AGUGG-GA-GC----UUCG----GC-AACGGUGAAAUA-CCACU-A-CUUUU-AA-C-GUUGUUUUAC-UUA
Spermatozopsis_exsultans          AGC-GCA-A-G-UAAC-C-U-G-//-GUGAAAUAACUUAAGAG-GUGUAGAAUA-AGUGG-GA-GC----UUCG----GC-AACGGUGAAAUA-CCACU-A-CUUUU-AA-C-GUUGUUUUAC-UUA
Stigeoclonium_helveticum          GGC-GCA-A-G-UAAC-C-U-G-//-GUGAAGCGACUUAAGAG-GUGUAGAAUA-AGUGG-GA-GC----UUCG----GC-GCCGGUGAAAUA-CCACU-A-CUUUU-AA-C-GUCGCUUUAC-UUA
Auxenochlorella_protothecoides    AGC-GCA-A-G-UAAC-C-U-G-//-GUGAAAUGACUUGGGAG-GUGUAGAAUA-AGUGG-GA-GUC---CUCG---GAC-GCCGGUGAAAUA-CCACU-A-CUCCC-AA-C-GUUAUUUUAC-UUA
Chromochloris_zofingiensis        AGC-GCA-A-G-UAAC-C-U-G-//-GUGAAAUAACUUAAGAG-GUGUAGAAUA-AGUGG-GA-GC----GCAA----GC-GCCGGUGAAAUA-CCACU-A-CUUUU-AA-C-GUUGUUUUAC-UUA
Chlorella_varibilis               AGC-GCA-A-G-UAAC-C-U-G-//-GUGAAAUGACUUAGGAG-GUGUAGAAUA-AGUGG-GA-GC----UUCG----GC-GCCGGUGAAAUA-CCACU-A-CUCUU/AA-C-GUUAUUUUAC-UUA
Coccomyxa_subellipsoidea          AGC-GCA-A-G-UAAC-C-U-G-//-GUGAAAUGACUUAAGGG-GUGUAGAAUA-AGUGG-GA-GC----UUCG----GC-GCCGGUGAAAUA-CCACU-A-CCUUU-AA-C-GUUAUUUUAC-UUA
Tetraselmis_striata               AGC-GUA-A-G-GAAA-C-U-A-//-GUGAAAUGACUUAAGAG-GUGUAGAAUA-AGUGG-GA-GC----UUCG----GC-GCCGGUGAAAUA-CCACU-A-CUUUU-AA-C-GUUAUUUUAC-UUA
Pedinomonas_minor                 GGC-GUA-A-U-GAAA-G-U-A-//-GUGAAAUGACUUGAGAG-GUGUAGAAUA-AGUGG-GA-GC----UUCG----GC-AACCGUGAAAUA-CCACU-A-CUUUU-AA-C-GUUAUUUUAC-UUA
Pycnococcus_provasolii            GGC-GCA-A-U-GAGA-G-U-A-//-GUGAAAUGACUUAGAAG-GUGUAGAAUA-AGUGG-GA-GC----UUCG----GC-GCCAGUGAAAUA-CCACU-A-CUUUU-AA-C-GUUAUUUUAC-UUA
Cymbomonas_tetramitiformis        GGC-GCA-A-G-UAAC-C-U-A-//-GUGAAAUGACUUAAGAG-GUGUAGAAUA-AGUGG-GA-GC----UCCG----GC-GACAGUGAAAUA-CCACU-A-CUUUU-GA-C-GUUAUUUUAC-UUA
Pyramimonas_parkeae               GGC-GCA-A-G-UAAC-C-U-A-//-GUGAAAUGACUUAGAAG-GUGUAGAAUA-AGUGG-GA-GC----UUCG----GC-GCCAGUGAAAUA-CCACU-A-CUUUU-AA-C-GUUAUUUUAC-UUA
Micromonas_bravo                  GGC-GCA-A-G-UAAC-C-U-G-//-GUGAAAUGACUUAAGAG-GUGUAGAAUA-AGUGG-GA-GC----UUCG----GC-GACAGUGAAAUA-CCACU-A-CUCUU-AA-C-GUUAUUUUAC-UUA
Bathycoccus_prasinos              GGC-GCA-G-G-UAAC-C-C-A-//-GUGAAAUGACUUAGGAG-GUGUAGAAUA-AGUGG-GA-GC----UUCG----GC-GAACGUGAAAUA-CCACU-A-CUCUU-AA-C-GUUAUUUUAC-UUA
Ostreococcus_tauri                GGC-GCA-A-G-UAAC-C-U-A-//-GUGAAACAACUUAGGAG-GUGUAGAAUA-AGUGG-GA-GC----UUCG----GC-GACUGUGAAAUA-CCACU-A-CUCUU-AA-C-GUUGUUUUAC-UUA
Mamiella_gilva                    GGC-GCA-A-G-UAAC-C-U-G-//-GUGAAAUGACUUAAGAG-GUGUAGAAUA-AGUGG-GA-GC----UUCG----GC-GACGGUGAAAUA-CCACU-A-CUUUU-AA-C-GUUAUUUUAC-UUA
Dolichomastix_tenuilepis          GGC-GCA-A-G-UAAC-C-U-G-//-GUGAAAUGACUUAGAAG-GUGUAGAAUA-AGUGG-GA-GC----UUCG----GC-GCCAGUGAAAUA-CCACU-A-CUUUU-AA-C-GUUAUUUUAC-UUA
Monomastix_opisthostigma          GGC-GCA-A-G-GAAC-C-U-G-//-GUGAAAUGACUUAAGAG-GUGUAGCAUA-AGUGG-GA-GC----UUCG----GC-GCCAGUGAAAUA-CCACU-A-CUUUU-AA-C-GUUAUUUUAC-UUA

Prasinoderma_coloniale            GGC-GCA-G-U-GAAA-G-C-G-//-GUGAAAUGAAUUAAGAG-GUGUAGAAUA-AGUGG-GA-GC----UUCG----GC-GCACGUGAAAUA-CCACU-A-CUUUU-AC-U-UUCUUUUUAC-UUA
Prasinoderma_singularis           GGC-GCA-G-U-GAAA-G-C-G-//-GUGAAAUGAAUUAAGAG-GUGUAGAAUA-AGUGG-GA-GC----UUCG----GC-GCACGUGAAAUA-CCACU-A-CUUUU-AC-U-UUCUUUUUAC-UUA
Prasinococcus_capsulatus          GGC-GCA-G-U-GAAA-G-C-A-//-GUGGAGAGAAUUAAGAG-GUGUAGAAUA-AGUGG-GA-GC----UCCG----GC-GCAAGUGAAAUA-CCACU-A-CUUUU-AC-U-UUCUUUUUAC-UUA
Verdigellas_peltata               GGC-ACA-G-U-GAAA-G-C-G-//-GUGAAAUGAAUUAAGAG-GUGUAGUUUA-AGUGG-GA-GC----UUCG----GC-GCAAGUGAAAGA-CCACU-A-CUUUU-AC-U-UUCUUUUUAC-UUA

Cyanophora_paradoxa               CGC-GUA-A-U-GAAA-G-U-A-//-GUGAAAUGACUUAAGAG-GUGUAGAAUA-AGUGG-GA-GGU---GCAA---GCC-GAA-GUGAAAUA-CCACU-A-CUUUU-AA-U-GUUAUUUUAC-UUA
Glaucocystis_nostochinearum       UGC-GCA-A-U-GAAA-G-U-A-//-GUGAAAUGACUUAAGAG-GUGUAGAAUA-AGUGG-GA-GGC---GCAA---GCC-GAAAUUGAAAUA-CCACU-A-CUUUU-AA-U-GUUAUUUUAC-UUA
Gloeochaete_wittrockiana          CGC-GUA-G-U-AAAA-G-C-A-//-GUGAAAUGACUUAAGAG-GUGUAGAAUA-AGUGG-GA-GGC---GCAA---GCC-GAAAGUGAAAUA-CCACU-A-CUUUU-AA-U-GUUAUUUUAC-UUA
Cyanoptyche_gloeocystis           CAC-GUA-G-U-GAAA-G-C-A-//-GUGAAAUGACUUAAGAG-GUGUAGAAUA-AGUGG-GA-GGC---GCAA---GCC-GAAAGUGAAAUA-CCACU-A-CUUUU-AA-U-GUUAUUUUAC-UUA

Palmaria_palmata                  GGC-GCA-G-U-GAAA-G-C-G-//-GUGAAGCAAGUUAGGAG-GUGUAGAAUA-GGUGG-GA-GC----UUCG----GC-AACCGUGAAAUA-CCACU-A-CUCUU-AA-U-CUUGCUUUAC-UUA
Chondrus_crispus                  GGC-GAA-G-U-GAAA-G-C-G-//-GUGAAGCAAGUUGGGAG-GCGUAGCAUA-GGUGG-GA-GCG---CUUG---CGC-GUCCGUGAAAUA-CCACU-A-CUCCC-AG-U-CUUGCUUUAC-UUA
Porphyra_umbilicalis              CGC-GCA-A-U-GAAA-G-U-A-//-GUGAAAUGAGUUAGGAG-GUGUAGUCUA-AGAGG-GA-GGG---GCAA---CCC-GACCGUGAAAUA-CCUCU-A-CUCUU-AA-U-CUUGUUUUAC-UUA
Compsopogon_coeruleus             CGC-GCA-A-U-GAAA-G-U-G-//-GUGAAAUGAGUUAAGAG-GUGUAGCAUA-AGUGG-GA-GC----UCCG----GC-GCCAGUGAAAUA-CCACU-A-CUUUU-AA-U-CUUGUUUUAC-UUA
Porphyridium_aerugineum           CGC-GCA-A-U-GAAA-G-U-A-//-GUGAAACGAGUUAGGAG-GUGUAGCAUA-AGUGG-GA-GCC---GCAA---GGC-GCCAGUGAAAUA-CCACU-A-CUCUU-AA-U-CUUGUUUUAC-UUA
Rhodosorus_marinus                CGC-GUA-A-U-GAAA-G-U-A-//-GUGAAAUGAGUUAAGAG-GUGUAGAAUA-AGUGG-GA-GC----UCCG----GC-GACCGUGAAAUA-CCACU-A-CUUUU-AA-U-CUUGUUUUAC-UUA
Rhodella_maculata                 CGC-GUA-A-U-GAAA-G-U-A-//-GUGAAAUGAGUUAGAAG-GUGUAGCAUA-AGUGG-GA-GC----UUCG----GC-GUCCGUGAAAUA-CCACU-A-CUUUU-AA-U-CUUGUCUUAC-UUA
Cyanidioschyzon_merolae           UGC-GCA-A-C-GAAA-G-U-G-//-GUGAAACGAGUUAGGAG-GUGUAGCAUA-AGUGG-GA-GCCG--CUCG--CGGC-GAUCCUGAAAUA-CCACU-A-CUCCU-AG-U-CUUGUUUUAC-UUA
rRNA_HELICES                      D3']---[D4-]----[D4']--//[------G4---------(----------)-----(--)-----]----[-----(------------)-----(-)--------(-)-----G4'--]---
```

Streptophyta

Chlorophyta

Prasino-dermophyta

Glaucoplantae

Rhodoplantae

Viridiplantae
