# Supplementary material for: The genome of Prasinoderma coloniale unveils the existence of a third phylum within green plants
Source: Nat Ecol Evol. 2020 Jun 22;4(9):1220–31. doi: 10.1038/s41559-020-1221-7 (PMC7455551; doi:10.1038/s41559-020-1221-7)
Supplement: Supplementary file 4 — Taxonomic Acts and Revisions. [file 41559_2020_1221_MOESM4_ESM.zip › Taxonomic Acts and Revisions 1/Diagnoses_Prasinodermophyta_corrected.pdf]

Taxonomic Diagnoses

Prasinodermophyta B. Marin & Melkonian divisio nova
Diagnosis: Non-motile, coccoid green algae without organic scales, surrounded by a cell wall. Occurring as free floating single cells or loose cell colonies, or organized as attached macroscopic palmelloid thalli of up to 25 cm diameter composed of distantly spaced cells embedded in a common gelatinous matrix. Cytokinesis unequal. Marine algae. In the nuclear-encoded SSU rRNA, the third from last nucleotide is G instead of U; in the nuclear-encoded LSU rRNA, base pair 11 of Helix G4 [H2093] is UxC instead of U-A.
With two classes: **Prasinodermophyceae** and **Palmophyllophyceae**

Prasinodermophyceae B. Marin & Melkonian classis nova
Diagnosis: Marine, coccoid green algae, single-celled, or forming loose planktonic colonies; cells small, ca. 2- 6 µm, surrounded by a thick multi-layered cell wall, without pores. The terminal base pair of Helix 30 [H861] in the chloroplast-encoded SSU rRNA is U-A instead of C-G.

Prasinodermales B. Marin & Melkonian ordo novus
Diagnosis: Characters as described for the class Prasinodermophyceae.

Prasinodermaceae B. Marin & Melkonian familia nova
Diagnosis: Characters as described for the order Prasinodermales.
Type genus: ***Prasinoderma*** Hasegawa & Chihara in Hasegawa et al. (1996) Phycologia 35: p. 171
Type species: ***Prasinoderma coloniale*** Hasegawa & Chihara in Hasegawa et al. (1996) Phycologia 35: 171, Fig 4.

Palmophyllophyceae Leliaert et al. (2016) Scientific Reports 6:25367, pp. 9, 10, emend. B. Marin & Melkonian
Emended diagnosis: Cells coccoid, (sub)spherical, ca. 3-14 µm, surrounded by a thin cell wall perforated by pores. Occurring as free floating single cells, or forming attached macroscopic palmelloid thalli of up to 25 cm diameter composed of distantly spaced cells embedded in a common gelatinous matrix. In the chloroplast-encoded 23S rRNA, the terminal nucleotide of the spacer between Helices C1 and D1 [H533 and H579] is U instead of A.
With two orders: Palmophyllales and Prasinococcales.

Note: By providing an emended diagnosis, we exclude members of the Prasinodermophyceae from the class Palmophyllophyceae, in accordance with molecular phylogenetic trees.
Type genus: ***Palmophyllum*** Kützing (1845-1849) Tabulae Phycologicae I. Band: p. 23; Kützing (1849) Species Algarum: p. 231
Type species: ***Palmophyllum flabellatum*** Kützing (1845-1849) Tabulae Phycologicae I. Band: p. 23, Tab. 32, Fig. V

Prasinococcales Guillou et al. [nomen nudum; validated by Leliaert et al. (2016) Scientific Reports 6:25367, p. 9] emend. B. Marin & Melkonian
Emended diagnosis: Solitary, coccoid green algae, about 3-8 μm. Cells surrounded by a thin cell wall perforated by pores, surrounded by a thick gelatinous capsule. Planktonic marine algae. In the nuclear-encoded 18S rRNA, base pair 3 of Helix 50 [H1506] is C-G instead of U-A.
Note: The order Prasinococcales, as originally defined by Leliaert et al. (2016), contained *Prasinococcus* and *Prasinoderma*. Because our molecular phylogenies revealed that these genera are not sister taxa, we restrict the order Prasinococcales to *Prasinococcus*, and exclude *Prasinoderma* by providing an emended diagnosis.
Type family: **Prasinococcaceae** Leliaert et al. (2016) Scientific Reports 6:25367, p. 9
Type genus: ***Prasinococcus*** Miyashita & Chihara in Miyashita et al. (1993) J. Gen. & Appl. Microbiol. 39: 578
Type species: ***Prasinococcus capsulatus*** Miyashita & Chihara in Miyashita et al. (1993) J. Gen. & Appl. Microbiol. 39: 580, Figs 3, 4

Glaucoplantae B. Marin & Melkonian subregnum novum
Diagnosis: Eukaryotic algae with cyanelles, i.e. blue-green plastids with chlorophyll a and phycobilins, stabilized by a peptidoglycan wall between the two envelope membranes. Motile biflagellate or non-motile coccoid or colonial algae. With cytoplasmic starch. Freshwater algae. In the nuclear-encoded 18S rRNA, base pair 3 of Helix 20 [H505] is A○G instead of C-G.
Type family: **Glaucocystaceae** West (1904) A Treatise on the British Freshwater Algae. Cambridge, p. 317
Type genus: ***Glaucocystis*** Itzigsohn (1854) in Rabenhorst: Flora Europaea Algarum Aquae Dulcis et Submarinae. Sectio III (1868), p. 417

Type species: ***Glaucocystis nostochinearum*** Itzigsohn in Rabenhorst (1866), Algen Europas, Decaden 94/95, no. 1935; and in Rabenhorst: Flora Europaea Algarum Aquae Dulcis et Submarinae. Sectio III (1868), p. 417
