# Supplementary material for: The genome of Prasinoderma coloniale unveils the existence of a third phylum within green plants
Source: Nat Ecol Evol. 2020 Jun 22;4(9):1220–31. doi: 10.1038/s41559-020-1221-7 (PMC7455551; doi:10.1038/s41559-020-1221-7)
Supplement: Supplementary file 4 — Taxonomic Acts and Revisions. [file 41559_2020_1221_MOESM4_ESM.zip › Taxonomic Acts and Revisions 1/Legends to Supplementary Figures_NHS.docx]

**Legends to Supplementary Figures (Molecular Synapomorphies)**

**Suppl. Fig. NHS_Prasinodermophyta_18S_G4.** Synapomorphy support for the Prasinodermophyta in the nuclear-encoded rRNA operon, visualized as rRNA secondary structures. As shown in the alignments, both synapomorphies are unique (= non-homoplasious synapomorphies [**NHS**]), i.e., they did not evolve in other Plantae (no homoplasies). Thus, these synapomorphies are unambiguous molecular signatures for the new division Prasinodermophyta, and were therefore included in the taxonomic diagnosis.

**Suppl. NHS_Chloro+Strepto_D4_G4.** Synapomorphy support for the sister-group relationship of the ‘classical’ green phyla/divisions Chlorophyta and Streptophyta, to the exclusion of the Prasinodermophyta. The common ancestor of Chlorophyta and Streptophyta changed the second base pair of Helix D4 in the nuclear 28S rRNA by two compensatory mutations [towards G-C], whereas the Prasinodermophyta retained the plesiomorphic U-G base pair. Similarly, an unpaired, highly conserved nucleotide in Helix G4 was substituted to C in all Chlorophyta and Streptophyta, without known homoplasies.
