# Supplementary material for: The genome of Prasinoderma coloniale unveils the existence of a third phylum within green plants
Source: Nat Ecol Evol. 2020 Jun 22;4(9):1220–31. doi: 10.1038/s41559-020-1221-7 (PMC7455551; doi:10.1038/s41559-020-1221-7)
Supplement: Supplementary file 4 — Taxonomic Acts and Revisions. [file NHS_Prasinodermophyta_18S_G4.pdf › Taxonomic Acts and Revisions 1/NHS_Prasinodermophyta_18S_G4.pdf]

rRNA Synapomorphies of the **Prasinodermophyta**, to the exclusion of Chlorophyta, Streptophyta, Glaucoplantae and Rhodoplantae

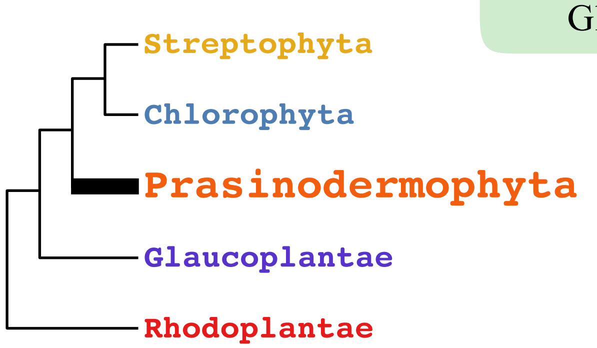

28S rRNA - Helix G4 [H2093]

bp 11: U-A ==> U x **C**

NHS (non homoplasious synapomorphy)

18S rRNA - terminal spacer after Helix 50 [H2093]

third from last nucleotide:

U ==> **G**

NHS (non homoplasious synapomorphy)

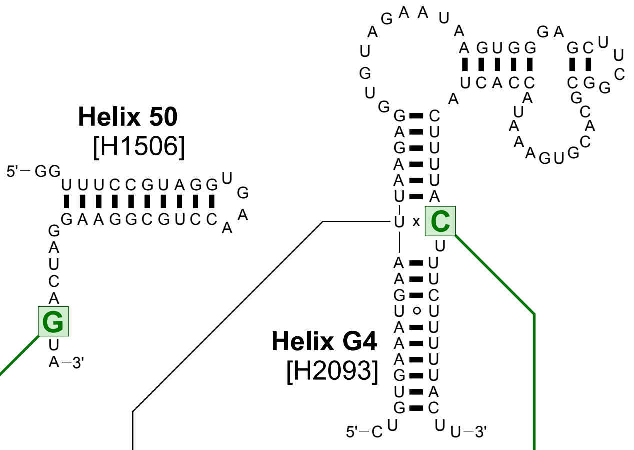


```
Prasinoderma_coloniale            -U-UUCCGU-AGG-UGAA-CCUGCGGAAG-GAUCA-G-UA-CCA-//-GUGAAAUGAAUUAAGAG/AGUGG-GA-GC----UUCG----GC/CCACU-A-CUUUU-A-C-U-UUCUUUUUAC-UUA
Prasinoderma_singularis           -U-UUCCGU-AGG-UGAA-CCUGCGGAAG-GAUCA-G-UA-CCA-//-GUGAAAUGAAUUAAGAG/AGUGG-GA-GC----UUCG----GC/CCACU-A-CUUUU-A-C-U-UUCUUUUUAC-UUA
Prasinococcus_capsulatus          -U-UCCCGU-AGG-UGAA-CCUGCGGGAG-GAUCA-G-UU-UUG-//-GUGGAGAGAAUUAAGAG/AGUGG-GA-GC----UCCG----GC/CCACU-A-CUUUU-A-C-U-UUCUUUUUAC-UUA
Verdigellas_peltata               -U-UUCCGU-AGG-UGAA-CCUGCGGAAG-GAUCA-G-UU-GAA-//-GUGAAAUGAAUUAAGAG/AGUGG-GA-GC----UUCG----GC/CCACU-A-CUUUU-A-C-U-UUCUUUUUAC-UUA

Arabidopsis_thaliana              -U-UUCCGU-AGG-UGAA-CCUGCGGAAG-GAUCA-U-UG-UCG-//-GUGAAAUGACUUGAGAG/AGUGG-GA-GC----UUCG----GC/CCACU-A-CUUUU-A-A-C-GUUAUUUUAC-UUA
Pinus_taeda                       -U-UACCGU-AGG-UGAA-CCUACGGUAG-GAUCA-U-UG-nnn-//-GUGAAAUGACUUGAGAG/AGUGG-GA-GCCG--AUUU--UGGC/CCACU-A-CUUUU-A-A-C-GUUAUUUUAC-UUA
Huperzia_selago                   -U-UUCCGU-AGG-UGAA-CCUGCGGAAG-GAUCA-U-UG-ACA-//-GUGAAAUGACUUGAGAG/AGUGG-GA-GC----UCCG----GC/CCACU-A-CUUUU-A-A-C-GUUAUUUUAC-UUA
Polytrichum_juniperinum           -U-UUCCGU-AGG-UGAA-CCnnnnnnnn-nnnnn-n-nn-nnn-//-GUGAAAUGACUUAAGAG/AGUGG-GA-GC-----/------GC/CCACU-A-CUUUU-A-A-C-GUUAUUUUAC-UUA
Marchantia_polymorpha             -U-UUCCGU-AGG-UGAA-CCUGCGGAAG-GAUCA-U-UG-nnn-//-GUGGAAUGACUCAAGAG/AGUGG-GA-GGUCC-GUGA-GGGCC/CCACU-A-CUUUU-G-A-C-GUUAUUUUAC-UUA
Nothoceros_vincentianus           -U-UUCCGU-AGG-UGAA-CCUGCGGAAG-GAUCA-U-UG-ACA-//-GUGAAAUGACUUAAGAG/AGUGG-GA-GCC---GUA----GGC/CCACU-A-CUUUU-A-A-C-GUUAUUUUAC-UUA
Mesotaenium_endlicherianum        -U-nnnnnn-nnn-nnnn-nnnnnnnnnn-nnnnn-n-nn-nnn-//-GUGAAAUGACUUAAGAG/AGUGG-GA-GGC---UUU----GCC/CCACU-A-CUUUU-A-A-C-GUUAUUUUAC-UUA
Cosmarium_ochthodes               -U/UUCCGU-AGG-UGAA-CCUGCGGAAG-GAUCA-U-UG-ACA-//-GUGAAAUGACUCAGGAG/AGUGG-GA-GG----GUGA----CC/CCACU-A-CUCUU-G-A-C-GUUAUUUUAC-UUA
Coleochaete_scutata               -U-UUCCGU-AGG-UGAA-CCUGCGGAAG-GAUCA-U-UG-ACA-//-GUGAAAUGACUUGAGAG/AGUGG-GA-GC----UCCG----GC/CCACU-A-CUUUC-A-A-C-GUUAUUUUAC-UUA
Chaetosphaeridium_globosum        -U-UUCCGU-AGG-UGAA-CCUGCGGAAG-GAUCA-U-UG-ACA-//-GUGAAAUGACUUAAGAG/AGUGG-GA-GC----UCCG----GC/CCACU-A-CUUUU-A-A-C-GUUAUUUUAC-UUA
Chara_braunii                     -U-UUCCGU-AGG-UGAA-CCUGCGGAAG-GAUCA-U-UG-ACA-//-GUGAAAUGACUUGAGAG/AGUGG-GA-GCC---UUAC---GGC/CCACU-A-CUUUU-A-A-C-GUUAUUUUAC-UUA
Klebsormidium_nitens              -U-UUCCGU-AGG-UGAA-CCUGCGGAAG-GAUCA-U-UG-ACA-//-GUGAAAUGACUUAAGAG/AGUGG-GA-GC----UUCG----GC/CCACU-A-CUUUU-A-A-C-GUUAUUUUAC-UUA
Chlorokybus_atmophyticus          -U-UUCCGU-AGG-UGAA-CCUGCGGAAG-GAUCA-U-UG-ACA-//-GUGAACUGGAUUGGGAG/AGUGG-GA-GC----UUCG----GC/CCACU-A-CUCCC-A-A-C-UCCGGUUUGC-UUA
Spirotaenia_condensata            -U-UUCCGU-AGG-UGAA-CCUGCGGAAG-GAUCA-U-UG-ACA-//-GUGAACUGGAAUGGGGG/AGUGG-GA-GC----UUCG----GC/CCACU-A-CCCCU-A-U-C-UCCGGUUUGC-UUA
Mesostigma_viride                 -U-UUCCGU-AGG-UGAA-CCUGCGGAAG-GAUCA-U-UA-CCG-//-GUGAACUGGAUCGGGAG/AGUGG-GA-GC----UCGC----GC/CCACU-A-CUCCC-G-A-C-UCCGGUUUGC-UUA

Ulva_mutabilis                    -U-CUCCGU-AGG-UGAA-CCUGCGGAGG-GAUCA-U-UG-AAA-//-GUGAAAUGACUUGAGAG/AGUGG-GA-GC----UUCG----GC/CCACU-A-CUUUU-A-A-C-GUUAUUUUAC-UUA
Chlamydomonas_reinhardtii         -U-UUCCGU-AGG-UGAA-CCUGCGGAAG-GAUCA-U-UG-nnn-//-GUGAAAUAACUUAAGAG/AGUGG-GA-GC----UUCG----GC/CCACU-A-CUUUU-A-A-C-GUUGUUUUAC-UUA
Volvox_carteri                    -U-UUCCGU-AGG-UGAA-CCUGCGGAAG-GAUCA-U-UG-AAU-//-GUGAAAUAACUUAAGAG/AGUGG-GA-GC----UUCG----GC/CCACU-A-CUUUU-A-A-C-GUUGUUUUAC-UUA
Spermatozopsis_exsultans          -U-UUCCGU-AGG-UGAA-CCUGCGGAAG-GAUCA-U-UG-AAU-//-GUGAAAUAACUUAAGAG/AGUGG-GA-GC----UUCG----GC/CCACU-A-CUUUU-A-A-C-GUUGUUUUAC-UUA
Stigeoclonium_helveticum          -U-UUCCGU-AGG-UGAA-CCUGCGGAAG-GAUCA-U-UG-AAU-//-GUGAAGCGACUUAAGAG/AGUGG-GA-GC----UUCG----GC/CCACU-A-CUUUU-A-A-C-GUCGCUUUAC-UUA
Auxenochlorella_protothecoides    -U-UUCCGU-AGG-UGAA-CCUGCGGAAG-GAUCA-U-UG-AUG-//-GUGAAAUGACUUGGGAG/AGUGG-GA-GUC---CUCG---GAC/CCACU-A-CUCCC-A-A-C-GUUAUUUUAC-UUA
Chromochloris_zofingiensis        -U-UUCCGU-AGG-UGAA-CCUGCGGAAG-GAUCA-U-UG-AAU-//-GUGAAAUAACUUAAGAG/AGUGG-GA-GC----GCAA----GC/CCACU-A-CUUUU-A-A-C-GUUGUUUUAC-UUA
Chlorella_varibilis               -U-UUCCGU/AGG-UGAA-CCUGCGGAAG-GAUCA-U-UG-AAU-//-GUGAAAUGACUUAGGAG/AGUGG-GA-GC----UUCG----GC/CCACU-A-CUCUU/A-A-C-GUUAUUUUAC-UUA
Coccomyxa_subellipsoidea          -U-UUCCGU-AGG-UGAA-CCUGCGGAAG-GAUCA-U-UG-CAU-//-GUGAAAUGACUUAAGGG/AGUGG-GA-GC----UUCG----GC/CCACU-A-CCUUU-A-A-C-GUUAUUUUAC-UUA
Tetraselmis_striata               -U-UUCCGU-AGG-UGAA-CCUGCGGAAG-GAUCA-U-UG-AAU-//-GUGAAAUGACUUAAGAG/AGUGG-GA-GC----UUCG----GC/CCACU-A-CUUUU-A-A-C-GUUAUUUUAC-UUA
Pedinomonas_minor                 -U-CUCCGU-AGG-UGAA-CCUGCGGAGG-GAUCA-U-UG-AAU-//-GUGAAAUGACUUGAGAG/AGUGG-GA-GC----UUCG----GC/CCACU-A-CUUUU-A-A-C-GUUAUUUUAC-UUA
Pycnococcus_provasolii            -U-UUCCGU-AGG-UGAA-CCUGCGGAAG-GAUCA-U-UA-ACU-//-GUGAAAUGACUUAGAAG/AGUGG-GA-GC----UUCG----GC/CCACU-A-CUUUU-A-A-C-GUUAUUUUAC-UUA
Cymbomonas_tetramitiformis        -U-UUCCGU-AGG-UGAA-CCUGCGGAAG-GAUCA-U-UG-CUA-//-GUGAAAUGACUUAAGAG/AGUGG-GA-GC----UCCG----GC/CCACU-A-CUUUU-G-A-C-GUUAUUUUAC-UUA
Pyramimonas_parkeae               -U-UUCCGU-AGG-UGAA-CCUGCGGAAG-GAUCA-U-UG-CAA-//-GUGAAAUGACUUAGAAG/AGUGG-GA-GC----UUCG----GC/CCACU-A-CUUUU-A-A-C-GUUAUUUUAC-UUA
Micromonas_bravo                  -U-UUCCGU-AGG-UGAA-CCUGCGGAAG-GAUCA-U-UA-CGG-//-GUGAAAUGACUUAAGAG/AGUGG-GA-GC----UUCG----GC/CCACU-A-CUCUU-A-A-C-GUUAUUUUAC-UUA
Bathycoccus_prasinos              -U-UUCCGU-AGG-UGAA-CCUGCGGAAG-GAUCA-U-UA-CAA-//-GUGAAAUGACUUAGGAG/AGUGG-GA-GC----UUCG----GC/CCACU-A-CUCUU-A-A-C-GUUAUUUUAC-UUA
Ostreococcus_tauri                -U-UUCCGU-AGG-UGAA-CCUGCGGAAG-GAUCA-U-UA-UGG-//-GUGAAACAACUUAGGAG/AGUGG-GA-GC----UUCG----GC/CCACU-A-CUCUU-A-A-C-GUUGUUUUAC-UUA
Mamiella_gilva                    -U-UUCCGU-AGG-UGAA-CCUGCGGAAG-GAUCA-U-UA-CGG-//-GUGAAAUGACUUAAGAG/AGUGG-GA-GC----UUCG----GC/CCACU-A-CUUUU-A-A-C-GUUAUUUUAC-UUA
Dolichomastix_tenuilepis          -U-UUCCGU-AGG-UGAA-CCUGCGGAAG-GAUCA-U-UA-GCU-//-GUGAAAUGACUUAGAAG/AGUGG-GA-GC----UUCG----GC/CCACU-A-CUUUU-A-A-C-GUUAUUUUAC-UUA
Monomastix_opisthostigma          -U-UUCCGU-AGG-UGAA-CCUGCGGAAG-GAUCA-A-UA-CCG-//-GUGAAAUGACUUAAGAG/AGUGG-GA-GC----UUCG----GC/CCACU-A-CUUUU-A-A-C-GUUAUUUUAC-UUA

Cyanophora_paradoxa               -U-UUCCGU-AGG-UGAA-CCUGCGGAAG-GAUCA-U-UA-ACA-//-GUGAAAUGACUUAAGAG/AGUGG-GA-GGU---GCAA---GCC/CCACU-A-CUUUU-A-A-U-GUUAUUUUAC-UUA
Glaucocystis_nostochinearum       -U-UUCCGU-AGn-nnnn-nnnnnnnnnn-nnnnn-n-nn-nnn-//-GUGAAAUGACUUAAGAG/AGUGG-GA-GGC---GCAA---GCC/CCACU-A-CUUUU-A-A-U-GUUAUUUUAC-UUA
Gloeochaete_wittrockiana          -U-UUCCGU-AGG-UGAA-CCUGCGGAAG-GAUCA-U-UA-AAU-//-GUGAAAUGACUUAAGAG/AGUGG-GA-GGC---GCAA---GCC/CCACU-A-CUUUU-A-A-U-GUUAUUUUAC-UUA
Cyanoptyche_gloeocystis           -U-UUCCGU-AGn-UnnA-CCUGCAGAAG-GAUCA-n-nn-nnn-//-GUGAAAUGACUUAAGAG/AGUGG-GA-GGC---GCAA---GCC/CCACU-A-CUUUU-A-A-U-GUUAUUUUAC-UUA

Palmaria_palmata                  -U-UUCCGU-AGG-UGAA-CCUGCGGAAG-GAUCA-U-UC-nnn-//-GUGAAGCAAGUUAGGAG/GGUGG-GA-GC----UUCG----GC/CCACU-A-CUCUU-A-A-U-CUUGCUUUAC-UUA
Chondrus_crispus                  -U-UUCCGU-AGG-UGAA-CCUGCGGAAG-GAUCA-U-UC-GUA-//-GUGAAGCAAGUUGGGAG/GGUGG-GA-GCG---CUUG---CGC/CCACU-A-CUCCC-A-G-U-CUUGCUUUAC-UUA
Porphyra_umbilicalis              -U-UUCCGU-AGG-UGAA-CCUGCGGAAG-GAUCA-U-UC-ACA-//-GUGAAAUGAGUUAGGAG/AGAGG-GA-GGG---GCAA---CCC/CCUCU-A-CUCUU-A-A-U-CUUGUUUUAC-UUA
Compsopogon_coeruleus             -U-UUCCGU-AGG-UGAA-CCUGCGGAAG-GAUCA-U-UC-ACG-//-GUGAAAUGAGUUAAGAG/AGUGG-GA-GC----UCCG----GC/CCACU-A-CUUUU-A-A-U-CUUGUUUUAC-UUA
Porphyridium_aerugineum           -U-CUCCGU-AGG-UGAA-CCUGCGGAGG-GAUCA-U-UC-nnn-//-GUGAAACGAGUUAGGAG/AGUGG-GA-GCC---GCAA---GGC/CCACU-A-CUCUU-A-A-U-CUUGUUUUAC-UUA
Rhodosorus_marinus                -U-UUCCGU-AGG-UGAA-CCUGCGGAAG-GAUCA-U-UC-ACA-//-GUGAAAUGAGUUAAGAG/AGUGG-GA-GC----UCCG----GC/CCACU-A-CUUUU-A-A-U-CUUGUUUUAC-UUA
Rhodella_maculata                 -U-UUCCGU-AGG-UGAA-CCUGCGGAAG-GAUCA-U-UC-ACA-//-GUGAAAUGAGUUAGAAG/AGUGG-GA-GC----UUCG----GC/CCACU-A-CUUUU-A-A-U-CUUGUCUUAC-UUA
Cyanidioschyzon_merolae           -U-UUCCGU-AGG-UGAA-CCUGCGGAAG-GAUCA-U-UC-nnn-//-GUGAAACGAGUUAGGAG/AGUGG-GA-GCCG--CUCG--CGGC/CCACU-A-CUCCU-A-G-U-CUUGUUUUAC-UUA
rRNA_HELICES                      [--50--------]----[-----50'--]---------------//[---G4------------/-----(--)-----]----[-----/-----(-)---------(-)----G4'---]---
```

Prasino-dermophyta

Streptophyta

Chlorophyta

Viridiplantae

Glaucoplantae

Rhodoplantae
