# Supplementary material for: The genome of Prasinoderma coloniale unveils the existence of a third phylum within green plants
Source: Nat Ecol Evol. 2020 Jun 22;4(9):1220–31. doi: 10.1038/s41559-020-1221-7 (PMC7455551; doi:10.1038/s41559-020-1221-7)
Supplement: Supplementary file 4 — Taxonomic Acts and Revisions. [file 41559_2020_1221_MOESM4_ESM.zip › Taxonomic Acts and Revisions 1/Table NHS Prasinodermophyta.doc]

**Table.** Synapomorphy support in nuclear and plastid-encoded ribosomal RNA operons for the new viridiplant division **Prasinodermophyta**, the classes **Prasinodermophyceae**, **Palmophyllophyceae** (emended; with two orders), and **Pyramimonadophyceae**, and the subkingdom **Glaucoplantae**. Nomenclature of rRNA secondary structures after (1) The European Ribosomal RNA Database (<http://web.archive.org/web/20110209200225/http://bioinformatics.psb.ugent.be/webtools/rRNA>), and (2) [nomenclature in brackets] the Gutell Lab CRW site (<http://www.rna.ccbb.utexas.edu/>). Unique molecular signatures that showed no homoplasies (i.e. parallelisms in other Viridi-, Glauco- or Rhodoplantae, or reversals) were flagged as **NHS** (**N**on-**H**omoplasious **S**ynapomorphy).

| Taxon/character | Evolutionary change | Characterization |
| --- | --- | --- |
| **Chlorophyta + Streptophyta excluding the Prasinodermophyta** |  |  |
| **28S rRNA** - Helix D4 [H628]: bp 2 | U-G ==> **G-C** | homoplasy in Chlorophyta: reversal to U-G in Pedinomonadales and Pseudoscourfieldiales  autapomorphic changes in e.g. *Mesostigma* (GxG), liverworts (A-U), *Ignatius* (A-U) |
| **28S rRNA** - reverse strand of Helix G4 [H2093]: unpaired nt between bp 10 and 11 | U ==> **C** | **C** = **NHS** |
| Division **Prasinodermophyta** |  |  |
| **18S rRNA** - 2-nt-spacer between Helices 24-25 [H655-H673]: nt 2 | U ==> **C** | parallelisms: C in *Roya obtusa* [Zygnematophyceae], *Pseudendoclonium arthopyreniae*, *Blidingia* [Ulvophyceae], *Chaetophora elegans* and *Desmotetra* [Chlorophyceae] |
| **18S rRNA** - terminal spacer after Helix 50 [H1506]: third from last nt | U ==> **G** | **G** = **NHS** |
| **28S rRNA** - Helix G4 [H2093]: bp 11 | U-A ==> Ux**C** | **C** = **NHS** |
| Class **Prasinodermophyceae *** |  |  |
| **18S rRNA** - Helix 15 [H339]: bp 2 | AxA ==> A-**U** | parallelism: A-U in *Galdieria* [Rhodoplantae] |
| **16S rRNA** - Helix 30 [H861]: bp 2 | C-G ==> **U-A** | **U-A** = **NHS** (few viridiplants have U-G, e.g. *Netrium*, *Ephedra*) |
| **16S rRNA** - 5-nt-spacer Helices 30-28 [H861-H821]: nt 2 [nt 870] | U ==> **G** | parallelism: G in *Choricystis parasitica* [Chlorophyta] |
| **23S rRNA** - forward strand of Helix E19 [H1648]: unpaired nt 5 [nt 1652] | A ==> **U** | **U** = **NHS** |
| **23S rRNA** - Helix G10: bp 5 [H2290: bp 3] | U-A ==> **A-U** | parallelism: A-U in *Pterosperma* [Chlorophyta] |
| **23S rRNA** - nucleotide before Helix G12 [nt 2322 before H2323] | A ==> **C** | **C** = **NHS** |
| Class **Palmophyllophyceae** emend. [including *Prasinococcus*, but excluding *Prasinoderma*] |  |  |
| **5.8S rRNA** - 2 nt before Helix B1 [H15] | A ==> **U** | parallelism: U in *Selaginella* [embryophytes] |
| **28S rRNA** - Helix D14: bp 6 [H837a: bp 7] | C-G ==> **U-A** | parallelism: U-A in Pseudoscourfieldiales [Chlorophyta] |
| **16S rRNA** - Helix 8 : bp 6 [H122: bp 7] | G-C ==> **A-U** | parallelism: A-U in *Pterosperma* [Chlorophyta] |
| **23S rRNA** - spacer Helices C1-D1 [H533-H579]: last nt [nt 578] | A ==> **U** | **U** = **NHS** |
| Order **Palmophyllales *** |  |  |
| **18S rRNA** - Helix 13 [H289]: bp 2 | G-C ==> **U-A** | **U-A** = **NHS** |
| **18S rRNA** - Helix 14 [H316]: bp 2 | G-C ==> **U-A** | **U-A** = **NHS** |
| **18S rRNA** - 8-nt-Loop of Helix 37 [H996]: nt 6 | A ==> **G** | **G** = **NHS** |
| **16S rRNA** - Helix 19: bp 1 [H500: bp 2] | C-G ==> **U**-G | **U**-G = **NHS** |
| **tRNA_Ile (GAU)** - acceptor stem: 1st and 2nd base pairs | G-C ==> **A-U**  G-C ==> **C-G** | both pairs = **NHS** |
| **23S rRNA** - Helix D3 [H604]: bp 3 | U-G ==> **G-U** | **G-U** = **NHS** |
| **23S rRNA** - Helix H1 [H2630]: bp 2 | G-C ==> **A-U** | **A-U** = **NHS** |
| Order **Prasinococcales** emend. [excluding *Prasinoderma*] |  |  |
| **18S rRNA** - Helix 50 [H1506]: bp 3 | U-A ==> **C-G** | **C-G** = **NHS** |
| **28S rRNA** - Helix B21 [H406]: bp 5 | G-C ==> **C-G** | **C-G** = **NHS** |
| **28S rRNA** – terminal loop of Helix G7 [H2259]: nt 1 | U ==> **A** | **A** = **NHS** |
| **16S rRNA** - 6-nt-spacer Helices 7-8 [H112-H122]: nt 5 | A ==> **U** | **U** = **NHS** |
| **16S rRNA** - Helix P23_1 [H588]: bp 1 | G-C ==> **U-A** | **U-A** = **NHS** |
| **16S rRNA** - Helix 27 [H769]: bp 1 | G-C ==> **U-A** | **U-A** = **NHS** |
| **23S rRNA** - Helix D8 [H687]: fifth from last bp | U-G ==> **A-U** | **A-U** = **NHS** |
| **23S rRNA** - Helix G20 [H2588]: last bp | C-G ==> **U-A** | **U-A** = **NHS** |
| Class **Pyramimonadophyceae** |  |  |
| **28S rRNA** - Helix G18: bp 5 [H2520: bp 2524/2539] | G-C ==> **C-G** | parallelism: C-G in the Ulvophyceae |
| **28S rRNA** - spacer Helices G1-H1: nt 1 [Helix H2043: bp 2044/2624] | G ==> **U**  [A🞆G ==> A-**U**] | parallelism: U in clade VII **-** prasinophytes |
| Subkingdom **Glaucoplantae** |  |  |
| **18S rRNA** - Helix 16 [H367]: last but one bp | C-G ==> **G-C** | parallelism: G-C in *Rozella* [Fungi] |
| **18S rRNA** - Helix 20 [H505]: bp 3 | C-G ==> **A**🞆G | **A = NHS** |

***** Due to missing 28S rRNA sequence data of *Palmophyllum* and of strain MBIC10622 (*Prasinoderma* sp.), synapomorphies in the 28S rRNA were ignored for the taxa Prasinodermophyceae and Palmophyllales.
